# From antidote to multimodal adjunct: a systematic review of N-acetylcysteine in dogs and cats

**DOI:** 10.3389/fvets.2026.1959626

**Published:** 2026-09-16

**Authors:** Zsombor Tamás Tóth, Noémi Katalin Tarpataki, Melinda Nemes-Terényi, Ákos Jerzsele, Ádám Kerek

**Affiliations:** 1 Department of Internal Medicine, University of Veterinary Medicine Budapest, Budapest, Hungary; 2 Department of Pharmacology and Toxicology, University of Veterinary Medicine Budapest, Budapest, Hungary; 3 National Laboratory of Infectious Animal Diseases, Antimicrobial Resistance, Veterinary Public Health and Food Chain Safety, University of Veterinary Medicine Budapest, Budapest, Hungary

**Keywords:** acetylcysteine, antidote, cats, dogs, evidence synthesis, N-acetylcysteine, oxidative stress, pharmacokinetics

## Abstract

**Introduction:**

N-acetylcysteine (NAC) is used in small-animal medicine as an antidote, mucolytic, thiol donor, and cytoprotective adjunct, yet its evidentiary basis varies markedly among indications and between dogs and cats. We systematically evaluated species-specific pharmacokinetic, clinical, experimental, and *in vitro* evidence to distinguish established uses from extrapolated practice.

**Methods:**

A 104-record legacy evidence library was re-screened, and an update search of PubMed/MEDLINE, Europe PMC, Crossref-resolved publisher records, Google Scholar, selected Hungarian databases and journal archives, and backward/forward citations was completed through 1 August 2026. Eligible reports evaluated NAC in dogs or cats *in vivo*, or in canine- or feline-derived tissues, cells, or clinical isolates. Risk of bias was appraised with design-specific tools, and findings were synthesized narratively because interventions, models, doses, and outcomes were too heterogeneous for meta-analysis.

**Results:**

Seventy-one reports were included in the qualitative synthesis. The strongest evidence supported early NAC administration for feline and canine acetaminophen toxicosis. Controlled or clinically direct evidence also supported redox restoration in hospitalized dogs, adjunctive treatment of canine parvoviral enteritis, short-course intravenous treatment in cats with acute-on-chronic kidney disease and selected topical ophthalmic applications. Antibacterial and antibiofilm activity against canine otitis and infectious-keratitis pathogens was reproducible *in vitro*, and a small clinical study supported multicomponent topical therapy for superficial pyoderma; however, NAC-specific effects and antimicrobial interactions remained formulation dependent. Experimental cardiopulmonary, renal, hepatic, and diabetic-organ-protection studies yielded biologically coherent but clinically immature signals. Conversely, perioperative neuroprotection, doxorubicin cardioprotection, and nebulized treatment of feline asthma were neutral or unfavorable. Conventional aqueous NAC pharmacokinetics remain undefined in dogs, and many widely quoted dosing schedules originate from expert synthesis rather than dose-ranging trials.

**Discussion:**

NAC is therefore best regarded as an evidence-supported antidote and a context-dependent adjunct, not a generic antioxidant therapy. Future studies should prioritize canine pharmacokinetics, standardized formulations, clinically meaningful outcomes, and prospective validation of topical antimicrobial and renal indications.

## 1 Introduction

N-acetylcysteine (NAC) occupies an unusual position in companion-animal therapeutics. It is simultaneously an established antidote, a classical mucolytic, a precursor of reduced glutathione (GSH), a reactive-thiol compound, and an increasingly investigated antimicrobial and organ-protective adjunct. This pharmacological breadth is attractive in diseases characterized by oxidative injury, viscous secretions, electrophilic metabolites, microvascular dysfunction, or biofilm formation. It also creates a recurrent interpretive problem: mechanistic plausibility is often treated as if it were equivalent to clinical efficacy.

The core chemistry is well-described. NAC is an N-acetylated cysteine derivative whose free sulfhydryl group can participate in nucleophilic reactions and disulfide exchange. After deacetylation, the released cysteine can support GSH synthesis when cysteine availability becomes rate limiting. Direct reaction kinetics are favorable for selected oxidants and electrophiles, but comparatively slow for hydrogen peroxide and superoxide under physiological conditions; much of the antioxidant effect is therefore indirect and dependent on cellular uptake, deacetylation, substrate limitation, and an intact glutathione system (1–4). NAC may additionally modulate redox-sensitive inflammatory pathways and alter extracellular matrices through thiol-disulfide chemistry (5, 6).

These mechanisms are clinically relevant in dogs and cats, but the two species cannot be treated as interchangeable. Cats have restricted glucuronidation capacity and are exceptionally vulnerable to acetaminophen-induced methaemoglobinaemia and oxidative erythrocyte injury (7, 8). They also respond adversely to direct airway exposure: nebulized NAC increased airway resistance in an experimental feline asthma model (9). Conversely, the only controlled pharmacokinetic study of conventional aqueous NAC in a companion species was performed in cats, whereas conventional-formulation canine pharmacokinetics remain undefined (10, 11).

The evidence base has expanded beyond antidotal use. Recent studies have examined NAC in canine parvoviral enteritis, babesiosis and pancytopenia, feline chronic kidney disease, canine otitis, infectious keratitis, critical illness, and several induced disease models. At the same time, a 2026 narrative review summarized canine and feline pharmacokinetics and therapeutic effects, emphasizing the need for species-specific interpretation (12). A further synthesis is justified only if it moves beyond catalog-style description. The present review therefore uses explicit eligibility criteria, design-specific quality appraisal, and an evidence-maturity framework to answer three questions: (i) which canine and feline indications are supported by direct clinical evidence; (ii) which dosing practices are experimentally supported, consensus-derived, or extrapolated; and (iii) where do negative findings, species differences, and translational limitations constrain use?

## 2 Materials and methods

This systematic review was designed and reported in accordance with PRISMA 2020 and PRISMA-S; narrative synthesis followed the SWiM framework (13–15). No meta-analysis was planned because the anticipated evidence encompassed pharmacokinetic crossover studies, controlled clinical trials, observational reports, induced-disease animal experiments, and *in vitro* assays with non-comparable outcomes. The review protocol was developed before the update search but was not prospectively registered because the systematic review evolved from a pre-existing topic-curated evidence library rather than being initiated *de novo* as a prospectively registered review. The absence of prospective registration is acknowledged as a methodological limitation because it reduces the external verifiability of pre-specified review decisions. The completed search strategies are provided in Supplementary Table S1.

### 2.1 Review question and eligibility criteria

The review question was structured using an expanded PICOS framework (Table 1). The population comprised dogs and cats, as well as canine- or feline-derived tissues, primary cells, cell lines, secretions, and clinical microbial isolates. The intervention was NAC, acetylcysteine, or N-acetyl-L-cysteine administered alone or in a defined combination. Comparators included placebo, vehicle, no treatment, baseline, standard care, or an active comparator; uncontrolled safety reports and case series were retained when they supplied otherwise unavailable clinical information. Outcomes included pharmacokinetic variables, antioxidant and inflammatory biomarkers, clinical efficacy, organ function, microbiological activity, biofilm endpoints, adverse events, and drug interactions.

**Table 1:** Pre-specified PICOS eligibility framework.

PICOS element	Operational definition
Population/model	Dogs and cats in clinical or experimental *in vivo* studies; canine- or feline-derived tissues, cells, secretions, or clinical microbial isolates.
Intervention	N-acetylcysteine, acetylcysteine, or N-acetyl-L-cysteine alone or in a defined combination; any route, dose, concentration, or formulation.
Comparator	Placebo, vehicle, untreated control, baseline, standard care, or active comparator. Uncontrolled safety reports retained when uniquely informative.
Outcomes	Pharmacokinetics, redox or inflammatory biomarkers, clinical response, organ function, survival, microbiological and biofilm endpoints, adverse events, and interactions.
Study designs	Clinical trials, observational studies, case reports/series, experimental *in vivo* studies, PK/PD studies, and species-specific *in vitro* or *ex vivo* studies.
Exclusions	No direct NAC evaluation; no canine/feline link; secondary publication without original data; conference abstract without adequate methods; commercial or non-peer-reviewed material.

Eligible designs were randomized or non-randomized clinical studies, cohort or retrospective studies, case series and case reports, experimental *in vivo* studies, species-specific pharmacokinetic or pharmacodynamic studies, and *in vitro* or *ex vivo* experiments using canine or feline material. English- and Hungarian-language full-text publications from database inception through 1 August 2026 were eligible; publications available only in other languages were not included in the primary evidence synthesis. Human, rodent, and livestock studies were excluded from the primary evidence synthesis; selected non-veterinary mechanistic sources were retained only to explain chemistry or reporting methods and were clearly separated from the included evidence. Conference abstracts without adequate methods, commercial material, editorials without original data, and studies that merely mentioned NAC were excluded. The pre-specified eligibility framework is summarized in Table 1.

### 2.2 Information sources and search strategy

The evidence base was assembled in two stages. First, the 104-record legacy library underlying the source manuscript was re-screened in full. This library had originally been developed from PubMed- and Crossref-indexed literature using combinations of the core drug terms (“N-acetylcysteine,” “acetylcysteine,” or “NAC”) with species terms (“dog,” “canine,” “cat,” “feline,” “small animal,” or “veterinary”) and indication- or mechanism-related terms, including “hepatotoxicity,” “otitis externa,” “pancreatitis,” “spinal cord,” “asthma,” “cardioprotection,” “oxidative stress,” and “glutathione.” Although complete database-specific yields and search histories were not retained for the legacy library, its coverage was strengthened by full-text re-screening of all 104 records and by a deliberately broader update search. The update search included PubMed/MEDLINE and Europe PMC records, Crossref-resolved publisher pages, Google Scholar, and journal/publisher websites. The principal update concept block was (“N-acetylcysteine” OR acetylcysteine OR “N-acetyl-L-cysteine” OR NAC) AND (dog OR dogs OR canine OR canines OR beagle OR beagles OR cat OR cats OR feline OR felines OR “small animal” OR “small animals” OR “companion animal” OR “companion animals”). Hungarian-language retrieval used MTMT, MATARKA, HuVetA, the online archive of Magyar Állatorvosok Lapja, and the Acta Veterinaria Hungarica archive with (N-acetilcisztein OR acetilcisztein OR NAC) AND (kutya OR macska). Reference lists of included studies and relevant reviews were examined manually, and forward citation searching was used for sentinel pharmacokinetic, toxicological, and clinical studies. Thus, the update search, journal-specific retrieval, and bidirectional citation chasing were used both to identify recent studies and to detect potentially relevant reports absent from the legacy library. The complete reproducible concept strings and source-specific adaptations used for the update are provided in Supplementary Table S1.

The initial manuscript library contained 104 records. Twelve additional NAC-specific records not represented in that library were identified during the update and citation search. All 12 additional records met the pre-specified eligibility criteria and were retained in the qualitative synthesis; consequently, the 45 full-text exclusions arose from the 104-record legacy library. All 116 records were assessed at full text because the legacy library had already been topic-curated. Database-of-origin metadata were not recoverable for every legacy record; consequently, the PRISMA diagram reports the auditable consolidated-library workflow rather than retrospective per-database yields. This limitation is considered explicitly in the Discussion and Supplementary methods.

### 2.3 Study selection, data extraction, and synthesis

All candidate reports were reassessed against the pre-specified criteria at full text. Full-text eligibility assessment and data extraction were performed by ZTT and subsequently verified by MN-T. Evidence-maturity assignments were additionally reviewed by NKT and AK. Any discrepancies in eligibility, extracted data, or evidence classification were resolved by discussion and consensus among the reviewing authors. For each included report, data were extracted into a standardized evidence table covering species, design, population or experimental model, sample size where reported, NAC formulation, route, dose or concentration, comparator, follow-up, principal outcomes, adverse events, and study limitations. The primary synthesis unit was the report. Evidence was grouped by clinical domain and proximity to practice: direct clinical evidence from spontaneous disease, controlled experimental *in vivo* evidence, and *in vitro* or *ex vivo* evidence; these evidence settings are displayed separately in Table 2 to prevent publication counts from being interpreted as equivalent levels of clinical support. Dosing statements were assigned a provenance label—trial-tested, experimentally tested, consensus-derived, or extrapolated—rather than being presented as uniformly validated recommendations. Given the non-comparable interventions and endpoints, results were synthesized narratively and displayed as an evidence-maturity map; no quantitative pooling was undertaken.

**Table 2:** Evidence setting, maturity, and clinical interpretation by indication.

Indication	Evidence setting	Evidence base	Direction	Maturity	Clinical interpretation
Acetaminophen toxicosis	Clinical + experimental/PK	Controlled feline challenge studies; canine PK/toxicology; clinical reports.	Favorable	Established	Administer early; strongest evidence in cats, with direct canine support.
Hepatobiliary disease	Clinical observational + experimental *in vivo + in vitro*	Experimental microcirculation; hepatic GSH studies; consensus dosing.	Mixed	Preliminary	Plausible adjunct; non-toxicant dosing is not validated by dose-ranging trials.
Respiratory mucolysis	Clinical observational + experimental *in vivo*	Early canine clinical observations; ciliary and bronchoprovocation models; feline asthma model.	Mixed/harmful in feline inhalation	Preliminary	Avoid nebulized NAC in cats with reactive airway disease.
Corneal disease	Experimental *in vivo + in vitro/ex vivo*	Canine wound and collagenase models; tear-film study; pathogen susceptibility.	Favorable but concentration-dependent	Supported	Adjunct for selected melting or epithelial defects; not a stand-alone antimicrobial.
Hospitalized critical illness	Clinical + experimental *in vivo*	Randomized canine trial plus endotoxaemia and ventilation models.	Biochemical benefit; no consistent outcome benefit	Preliminary	Do not use solely because oxidative stress is present.
Canine parvoviral enteritis	Clinical, spontaneous disease	Two randomized or controlled clinical studies.	Favorable surrogate endpoints	Supported	Promising adjunct; survival and length-of-stay benefit unproven.
Canine babesiosis/pancytopenia	Clinical/translational, spontaneous disease	Three recent small clinical or translational studies.	Favorable	Preliminary	Hypothesis-generating; not antiparasitic monotherapy.
Feline acute-on-chronic CKD	Clinical, spontaneous disease	One double-blind placebo-controlled clinical trial.	Favorable	Supported	Requires independent replication; evidence concerns a 7-day IV course.
Canine otitis/biofilm	*In vitro*	Multiple *in vitro* isolate and biofilm studies.	Mixed; combination-specific	Experimental	High local concentrations are active; clinical cure and otic safety remain unknown.
Sialocele	Clinical retrospective, spontaneous disease	Retrospective canine case series.	Favorable	Preliminary	Potential low-morbidity adjunct; no comparative trial.
Neurological injury	Clinical + experimental *in vivo*	Two controlled canine studies and one uncontrolled protocol.	Neutral/unfavorable	Unsupported as tested	Single-dose neuroprotection not demonstrated.
Cardiac ischemia–reperfusion	Experimental *in vivo*	Multiple experimental canine models.	Mixed	Experimental	Some infarct/function benefit, strongly dependent on model and timing.
Doxorubicin cardiotoxicity	Experimental *in vivo*	Two independent canine studies.	Neutral for function/survival	Unsupported as tested	Does not replace dexrazoxane or monitoring.
Renal ischemia–reperfusion	Experimental *in vivo*	Two canine surgical models.	Favorable	Experimental	Mechanistically plausible; not evidence for routine canine CKD use.
Experimental diabetes	Experimental *in vivo;* common induced-disease platform	Ten related reports from a common chemically induced T1DM beagle experimental platform.	Favorable molecular endpoints	Experimental	Common experimental platform with no independent replication or spontaneous-disease clinical validation.

Evidence setting distinguishes studies in animals with spontaneous clinical disease from experimental *in vivo* models and *in vitro* or *ex vivo* evidence.

The 71 reports meeting the pre-specified eligibility criteria constituted the primary evidence dataset of the systematic review. The manuscript reference list contains 101 citations in total because it additionally includes 30 methodological, mechanistic, review, and contextual sources used to support the review framework and interpretation; these sources were not counted as studies included in the qualitative synthesis.

### 2.4 Risk of bias and evidence maturity

Randomized clinical studies were evaluated for sequence generation, allocation, blinding, incomplete outcome data, selective reporting, and other bias. Non-randomized intervention studies were considered with ROBINS-I principles (16); case reports and case series were assessed for diagnostic clarity, intervention description, follow-up, and alternative explanations. Experimental animal studies were appraised using the SYRCLE domains (17). *In vitro* and *ex vivo* studies were assessed using an adapted reliability framework derived from ToxRTool, emphasizing model identity, replicate structure, vehicle controls, concentration verification, endpoint validity, and completeness of reporting (18).

Because study designs were heterogeneous, no single pooled certainty score was assigned. Instead, each indication was classified using a review-specific evidence-maturity framework as established (replicated, species-specific clinical or antidotal evidence); supported (controlled clinical evidence or convergent direct evidence, but with remaining replication or translational limitations); preliminary (small, uncontrolled, mixed, or otherwise limited direct clinical evidence); experimental (predominantly induced-disease or *in vitro* evidence); or unsupported as tested (controlled evidence showing no meaningful benefit or clinically relevant harm). Direction of effect was categorized as favorable, mixed/context-dependent, neutral, or unfavorable. These evidence-maturity categories were descriptive and review-specific and were not intended to represent formal GRADE certainty-of-evidence ratings.

## 3 Results

The auditable evidence library comprised 116 unique records: 104 legacy records and 12 additional NAC-specific reports identified during the update. Because the source library was already topic-curated, all 116 reports underwent full-text eligibility assessment. Of the 104 legacy records, 59 met the eligibility criteria and 45 were excluded, most commonly because they were reviews or background mechanistic papers without original canine/feline NAC data, evaluated no direct NAC exposure, or used no eligible canine/feline model. All 12 additional records identified during the update met the eligibility criteria. Accordingly, the final qualitative synthesis comprised 71 reports: 59 from the legacy library and 12 newly identified reports (Figure 1). The newly identified reports broadened the evidence base across clinical or translational, controlled experimental, and *in vitro* or *ex vivo* study designs. These 71 reports represent the included primary evidence base and should be distinguished from the complete manuscript bibliography, which also contains methodological and contextual references that were not eligible for inclusion as primary studies. The evidence was dominated by dog-only studies; feline clinical evidence remained concentrated in acetaminophen toxicosis, pharmacokinetics, airway safety, and acute-on-chronic kidney disease. Publication dates ranged from 1980 to 2026.

**Figure 1:**
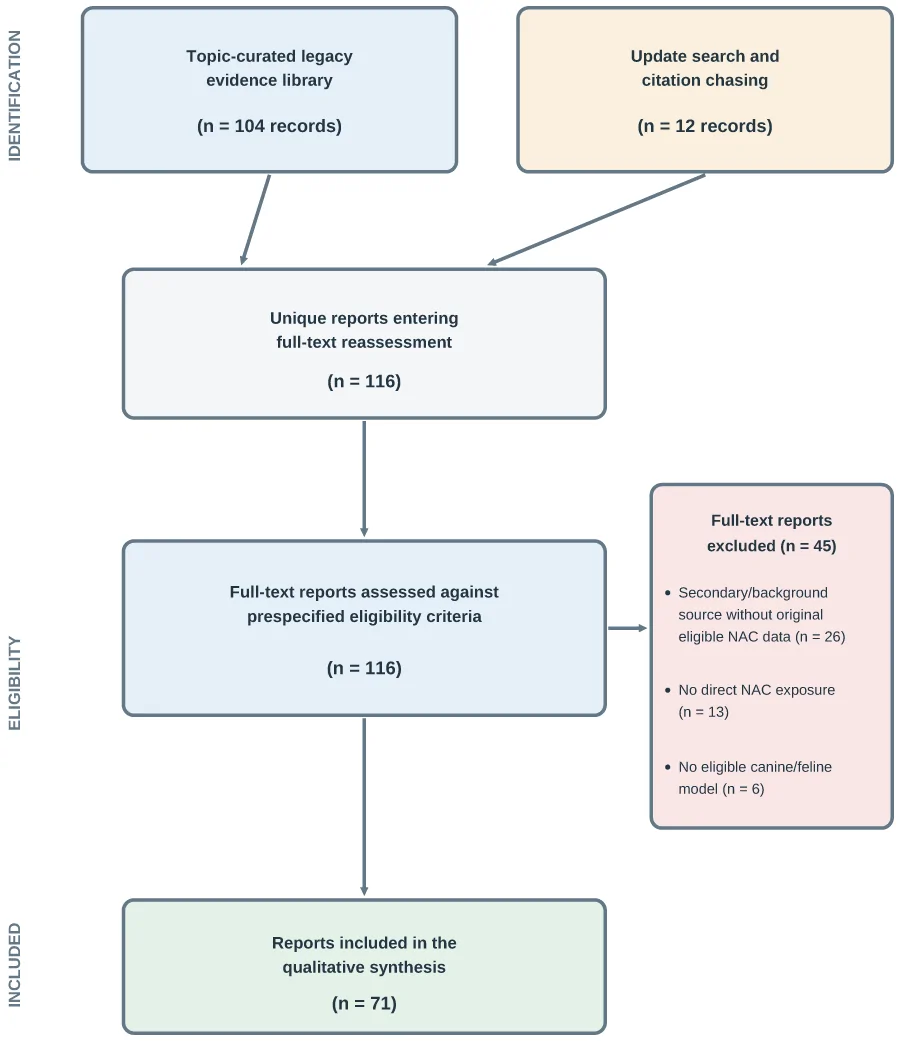
PRISMA 2020 flow diagram for evidence-library consolidation, full-text reassessment, eligibility assessment, and inclusion. The auditable evidence library comprised 104 legacy records and 12 additional records identified during the update search. Of the 104 legacy reports, 59 met the eligibility criteria and 45 were excluded; all 12 additional reports identified during the update met the eligibility criteria, yielding 71 reports in the final qualitative synthesis. PRISMA counts refer exclusively to reports evaluated for inclusion in the primary evidence synthesis and should not be equated with the 101 citations contained in the complete manuscript bibliography, which additionally includes methodological, mechanistic, review, and contextual sources. Because database-of-origin metadata were unavailable for some legacy records, the diagram reports consolidated rather than retrospective per-database yields.

The evidence landscape was highly asymmetric (Figure 2; Table 2). Acetaminophen toxicosis had the strongest species-specific foundation. Several newer indications—parvoviral enteritis, feline acute-on-chronic kidney disease, canine babesiosis, and local antimicrobial applications—were supported by direct but limited studies. Cardiovascular, renal ischemia-reperfusion, hepatic, neurological, and diabetic-organ-protection literatures were dominated by purpose-bred canine models rather than spontaneous disease.

**Figure 2:**
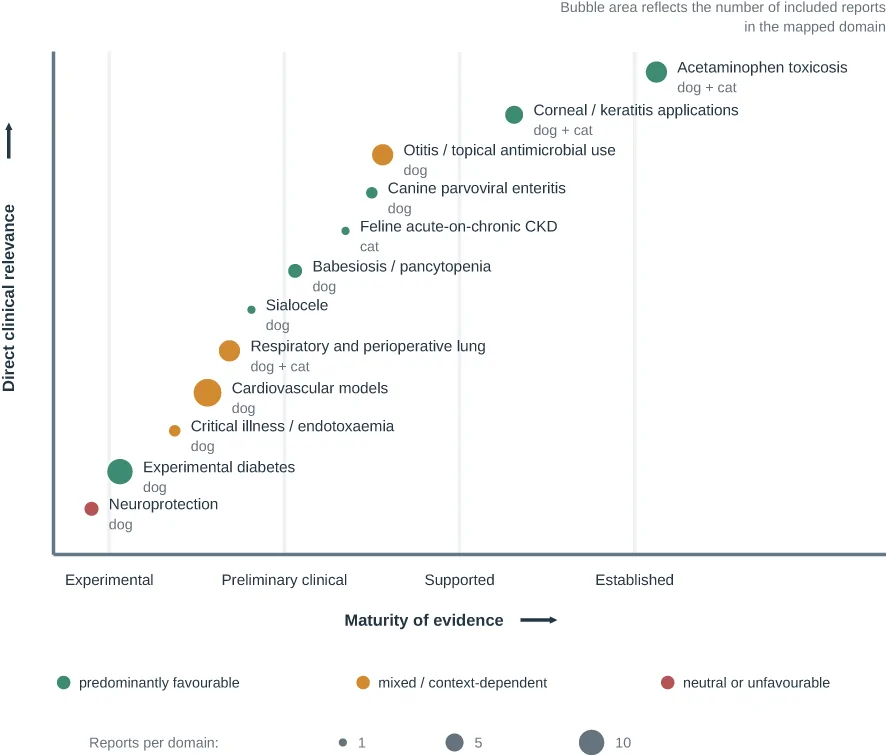
Evidence landscape by direct clinical relevance, maturity, and direction of effect. Bubble area reflects the number of included reports assigned to each mapped domain. Position reflects the overall evidence classification rather than a quantitative meta-analytic effect estimate. The experimental diabetes domain comprises 10 publications arising from a closely related experimental platform and should not be interpreted as representing 10 independent evidence streams.

All 71 primary reports constituting the qualitative evidence base are individually identified in Supplementary Table S2, with their study characteristics and evidence domain; the corresponding design-specific appraisal is provided in Supplementary Table S3. The complete report-level exclusion list and the primary reason assigned to each excluded report are provided in Supplementary Table S4.

### 3.1 Pharmacokinetics and disposition

The only crossover pharmacokinetic study of conventional aqueous NAC in companion animals enrolled six healthy cats given 100 mg/kg orally and intravenously. Mean oral bioavailability was 19.3%, and elimination was rapid after both routes, with mean terminal half-lives of approximately 0.78 h intravenously and 1.34 h orally (10). These data support the intuitive need for higher oral doses or repeated administration, but they do not establish therapeutic exposure targets for any disease.

No equivalent study of standard aqueous NAC was identified in dogs. A beagle study characterized liposomal formulations containing NAC or tocopherols after intravenous administration, primarily for formulation safety and disposition; its vehicle, formulation, and purpose limit direct translation to routine aqueous products (11). This absence is consequential: many canine schedules are repeated in formularies and reviews, yet their dosing interval has not been anchored to conventional-formulation canine exposure-response data.

NAC circulates as reduced free drug, disulfides, mixed disulfides, and protein-bound forms. Rapid deacetylation supplies cysteine, while oxidation, incorporation into thiol pools, and renal elimination contribute to clearance. Human pharmacokinetic work remains useful for chemical interpretation, but it should not be used to assign precise canine bioavailability or half-life values (19–21).

Route of administration should therefore be interpreted together with the limited species-specific pharmacokinetic evidence. In cats, oral administration of conventional aqueous NAC results in relatively low systemic bioavailability (19.3%) and a short terminal half-life (approximately 1.34 h), whereas intravenous administration provides immediate systemic exposure with a terminal half-life of approximately 0.78 h (10). These findings demonstrate substantial route-dependent differences in exposure but do not establish disease-specific target concentrations or optimal dosing intervals. When intravenous NAC is used, dilution, slow infusion, and monitoring are appropriate because infusion-related reactions can occur. In dogs, conventional aqueous NAC pharmacokinetics have not been adequately characterized; consequently, commonly used systemic regimens are based largely on toxicological studies, experimental protocols, and clinical consensus rather than species-specific exposure–response relationships. Local routes, including ophthalmic, otic, intracanalicular, and inhaled administration, produce tissue exposures that cannot be inferred from systemic pharmacokinetics and should be considered separately because efficacy and tolerability are concentration-, formulation-, and species-dependent. The principal veterinary regimens and their evidence provenance are summarized in Table 3.

**Table 3:** Veterinary NAC regimens, routes of administration, and evidence provenance reported in clinical trials, experimental studies, or expert syntheses.

Indication/context	Regimen reported in veterinary literature	Route	Evidence provenance	Key caution
Acetaminophen toxicosis	140 mg/kg loading, followed by 70 mg/kg every 6 h for 5–17 further doses	IV or PO	Controlled toxicology + longstanding clinical synthesis	Begin promptly; dilute and infuse IV doses slowly.
Alternative 20-h antidotal protocol	140 mg/kg loading; 50 mg/kg over 4 h; then 100 mg/kg over 16 h	IV infusion	Consensus-derived adaptation	Fluid volume and infusion reactions require monitoring.
Hospitalized ill dogs	Protocols designed to provide NAC during the first 48 h	PO/IV	Randomized clinical trial	Biochemical repletion did not improve survival.
Canine parvoviral enteritis	70 mg/kg once daily for 5 days in one trial	IV	Trial-tested	Adjunct to supportive care; small studies.
Canine babesiosis	70 mg/kg once daily for 5–14 days	PO	Pilot clinical studies	Relapse and survival effects unknown.
Feline acute-on-chronic CKD	70 mg/kg once daily for 7 days	IV	Double-blind clinical trial	Not evidence for chronic maintenance treatment.
Canine corneal epithelial healing	3% topical solution in experimental wounds	Topical ophthalmic	Controlled experimental study	Higher concentrations did not accelerate epithelial healing.
Corneal melting/collagenolysis	Commonly 5–10% solution at frequent initial intervals	Topical ophthalmic	Experimental inhibition + clinical consensus	Compound aseptically; monitor irritation and stromal stability.
Canine otitis/biofilm	Approx. 5–80 mg/mL depending on organism and biofilm state	Topical/*in vitro*	*In vitro* only	Interactions can be indifferent or antagonistic.
Canine sialocele	10% solution; median volume about 5 mL/gland in case series	Intracanalicular	Retrospective case series	Technique and anatomy limit generalization.
Experimental myocardial/organ protection	50–200 mg/kg bolus or infusion schedules	IV	Induced-disease models	Not routine clinical dosing; effect is model-specific.

These entries should be interpreted in the context of the limited species-specific pharmacokinetic evidence and are not a substitute for patient-specific prescribing.

### 3.2 Mechanistic evidence and biological plausibility

The central indirect mechanism is restoration of cysteine availability for GSH synthesis. This is most relevant when cysteine and GSH have been depleted by electrophilic metabolism, severe inflammation, or sustained oxidant burden. Clinically ill dogs and cats can have disturbed glutathione, cysteine, and ascorbate status, providing a biological substrate for supplementation (22). In a randomized investigator-blinded trial of 60 hospitalized ill dogs, NAC increased plasma cysteine and helped maintain erythrocyte glutathione, but did not significantly improve illness severity score or survival, illustrating the difference between target engagement and patient-level efficacy (23).

Direct scavenging is selective rather than universal. NAC reacts readily with hypochlorous acid, hydroxyl radicals, selected nitrogen species, and electrophiles; direct reaction with hydrogen peroxide and superoxide is comparatively slow at physiological concentrations (1, 2). Redox-sensitive modulation of NF-κB, NLRP3, and related pathways is pharmacologically plausible, but canine and feline pathway conservation does not prove that every clinical dose produces meaningful pathway inhibition (5, 24).

The free thiol can reduce disulfide crosslinks in mucin and other extracellular polymers. This explains mucolysis and may contribute to disruption of biofilm matrices or inhibition of corneal collagenolysis. Figure 3 integrates these mechanisms with routes of administration and species-specific constraints. Importantly, the same local chemistry can irritate hyper-reactive airways, and high concentrations may behave differently from systemic low-concentration exposure.

**Figure 3:**
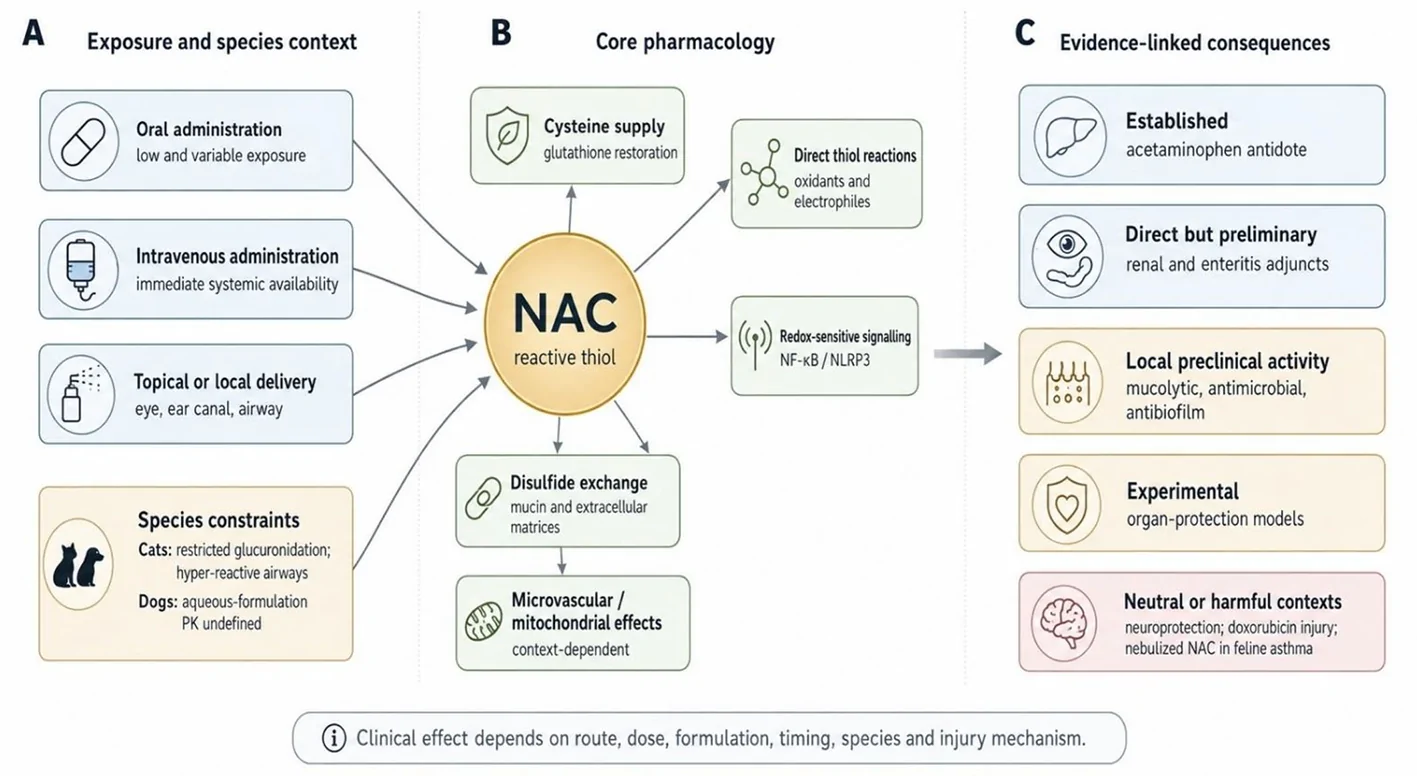
Mechanistic and translational framework for N-acetylcysteine in canine and feline medicine. **(A)** Route and species context determine exposure and local tolerability. **(B)** Principal mechanisms include cysteine delivery for glutathione synthesis, selective thiol chemistry, redox-sensitive signaling, and disulfide exchange. **(C)** Clinical effects range from established antidotal use to local mucolytic or antimicrobial activity and experimental organ protection.

### 3.3 Acetaminophen toxicosis and hepatobiliary use

Feline acetaminophen toxicosis remains the clearest indication. Controlled challenge studies demonstrated that NAC reduced methaemoglobinaemia, glutathione depletion, Heinz-body formation, and mortality, with greater protection when treatment began early (25–28). The effect is mechanistically coherent: cysteine provision supports GSH-dependent detoxification of reactive metabolites, while thiol chemistry may also contribute to redox recovery. Cats require urgent treatment because limited glucuronidation and erythrocyte susceptibility narrow the margin of safety.

Direct canine evidence is smaller but relevant. In beagles challenged with acetaminophen, an oral NAC regimen shortened acetaminophen half-life and increased total body clearance (29). Clinical canine reports describe recovery from methaemoglobinaemia and haemolysis within multimodal treatment, although causal attribution to NAC alone is not possible (30). Standard loading and maintenance schedules in Table 3 therefore rest on a combination of controlled toxicology, long clinical experience, and expert synthesis rather than modern dose-ranging trials (31, 32).

Evidence for non-acetaminophen hepatobiliary use is less mature. Dogs with naturally occurring hepatic disease can have depleted hepatic GSH (33), providing a rationale for supplementation. In obstructive jaundice, NAC improved portal and hepatic microcirculatory flow and increased hepatic GSH and ATP in an experimental canine model (34). In contrast, short-term NAC did not protect against biochemical or functional injury in a canine hepatic inflow-occlusion model (35). For canine biliary disease, 20% NAC reduced the viscosity of normal bile *in vitro*, but a similar effect was produced by water dilution, so a specific mucolytic advantage remains unproven (36).

### 3.4 Respiratory applications

The mucolytic rationale is chemically strong, but the veterinary clinical evidence remains limited. Early canine observations suggested symptomatic and gas-exchange improvement in chronic obstructive bronchitis treated with acetylcysteine-containing protocols, although the available report does not specify a body-weight-adjusted systemic NAC dose (37). Experimental work showed concentration-dependent effects on canine tracheal ciliary activity, with stimulation at lower exposure and ciliodepression at higher concentrations (38). In a controlled methacholine-challenge model, dogs received NAC intravenously as a 150 mg/kg loading dose followed by a continuous infusion of 20 mg/kg/h; this regimen improved arterial oxygenation and ventilation–perfusion matching, although the time course of bronchoconstriction was not altered (39).

Experimental canine studies also provide direct evidence for pulmonary antioxidant and anti-inflammatory effects of systemic NAC. During prolonged exposure to 100% oxygen, intravenous NAC (150 mg/kg loading dose followed by 20 mg/kg/h) attenuated increases in pulmonary vascular resistance, PaCO_2_, and lung wet weight, preserved dynamic compliance, delayed ventilation–perfusion abnormalities, and reduced pulmonary leukocyte accumulation and oedema (40). In a canine cardiopulmonary-bypass model, the same intravenous regimen improved oxygenation, increased pulmonary superoxide dismutase activity, reduced malondialdehyde accumulation and histological lung injury, and suppressed pulmonary TGF-β1 expression (41). In addition, intravenous NAC at 200 mg/kg prevented the loss of hypoxic pulmonary vasoconstriction during hemodilution, an effect interpreted as being at least partly related to modulation of reactive oxygen species (42). These findings support pulmonary antioxidant and anti-inflammatory activity in dogs, but they derive from controlled experimental injury models and should not be interpreted as evidence of clinical efficacy in spontaneous canine respiratory disease.

The clearest respiratory safety signal is feline. Endotracheal nebulization increased airway resistance in cats with experimental asthma (9). Systemic NAC and local nebulized NAC should therefore not be conflated: the former may be considered as an antioxidant adjunct in selected circumstances, whereas direct inhalation in cats with reactive airway disease is not supported and may be harmful. Human systematic evidence on mucus hypersecretion can explain the pharmacology but cannot override this species-specific result (43).

### 3.5 Ophthalmic applications

Topical NAC has two distinct ophthalmic rationales: inhibition of corneal proteolysis and direct antimicrobial activity. In experimentally created canine corneal wounds, 3% NAC accelerated epithelial healing, whereas 10% and 20% solutions did not improve healing relative to control; no concentration produced overt histological toxicity (44). In a corneal degradation model, concentration and comparator materially affected collagenase inhibition (45). By contrast, a study of normal canine tear-film gelatinase found only limited inhibition with 10% NAC, underscoring that anti-melting activity in a diseased cornea cannot be inferred from every assay (46).

Recent microbiological work broadened the rationale. NAC inhibited canine and feline infectious-keratitis isolates of *Staphylococcus pseudintermedius, Streptococcus canis*, and *Pseudomonas aeruginosa in vitro*, although required concentrations differed among organisms and direct clinical efficacy has not been tested (47). A 2025 comparison of commercially available antiseptics confirmed bactericidal NAC activity while showing that protein-rich media materially altered apparent efficacy (48). Taken together, topical 3–10% use is supported most strongly as an adjunct in selected corneal wounds or keratomalacia, not as a stand-alone substitute for antimicrobial therapy, debridement, or surgical stabilization.

### 3.6 Critical illness and infectious-disease adjuncts

NAC has repeatedly engaged redox endpoints in critical illness without consistently improving global outcome. In canine endotoxic shock, an infusion protocol improved oxygen delivery, extraction, lactate clearance, and tumor-necrosis-factor response without major hemodynamic deterioration (49). Continuous infusion during prolonged hyperoxia delayed pulmonary vascular and compliance abnormalities and reduced inflammatory lung injury (40); cardiopulmonary bypass and hemodilution models also yielded favorable physiological or histological findings (41, 42). These experiments support biological activity, but their high-dose, invasive settings differ from heterogeneous spontaneous disease.

The randomized trial in hospitalized ill dogs is therefore particularly informative: biochemical repletion occurred, but severity score and survival did not improve (23). This neutral clinical result argues against routine NAC administration solely because a patient is systemically ill. The background literature on oxidative stress in canine disease is extensive, including recent Hungarian reviews of pancreatitis and intestinal disease models, but redox disturbance alone is not an efficacy criterion (50–52).

Canine parvoviral enteritis provides a more focused signal. In a small non-blinded trial, 70 mg/kg NAC intravenously once daily for 5 days improved leukocyte recovery and oxidative-stress markers relative to supportive care (53). A later randomized outpatient study compared several antioxidant adjuncts and found that NAC reduced malondialdehyde, nitric oxide, and intestinal fatty-acid-binding protein, with improved leukocyte and neutrophil recovery, but survival benefit was not established (54). The consistency of biomarker direction across two clinical studies is encouraging, although both remain limited by sample size, co-interventions, and surrogate outcomes.

Three recent canine hemoprotozoan studies also reported favorable adjunctive signals. Oral NAC improved oxidative, hematological, acute-phase, hepatic, or renal markers when added to standard treatment for *Babesia gibsoni* infection, but the pilot designs were underpowered for relapse or survival (55, 56). A mixed preclinical-clinical study suggested improved marrow recovery in pancytopenic dogs with hemoprotozoan disease, but extrapolation from a cyclophosphamide rat model and incomplete control of confounding reduce certainty (57). These results should be treated as hypothesis-generating, not as proof that NAC is a primary antiparasitic or hematopoietic drug.

### 3.7 Renal indications

A 2026 double-blind placebo-controlled trial enrolled 50 cats with acute-on-chronic azotemic chronic kidney disease and compared seven daily intravenous doses of 70 mg/kg NAC with saline, in addition to fluid therapy. Creatinine, symmetric dimethylarginine, blood urea nitrogen, and urine protein-to-creatinine ratio improved more with NAC (58). This is the most direct renal evidence in companion animals, but it addresses short-term treatment during an exacerbation rather than stable chronic disease or long-term oral supplementation. Contemporary practice surveys confirm substantial variation in feline CKD management, reinforcing the need to replicate this result before routine adoption (59).

Canine evidence is experimental. NAC preserved glomerular filtration and endothelium-dependent renal vascular responses after inferior vena cava occlusion in dogs (60). In an autologous renal-transplantation model, a protocol combining NAC with AICAR improved graft function, but NAC was not tested alone (61). The current evidence therefore supports a plausible peri-ischaemic renal effect and a preliminary feline clinical indication, but not a generalized claim of nephroprotection across contrast exposure, stable CKD, or all causes of acute kidney injury. The toxicological context is also important: clinical work on grape and raisin poisoning emphasizes that canine acute kidney injury is mechanistically heterogeneous and often lacks a specific antidote (62).

### 3.8 Antimicrobial, antibiofilm, and antifungal activity

Canine otitis externa is the best-developed local antimicrobial field. NAC inhibited clinical otic isolates *in vitro*, generally at milligram-per-milliliter concentrations (63, 64). Established biofilms required substantially higher concentrations than planktonic organisms, with *P. aeruginosa* biofilms particularly tolerant (65). Combination effects were not uniformly favorable: NAC was commonly indifferent or antagonistic with enrofloxacin or gentamicin (66), showed little consistent synergy with ketoconazole or nystatin against *Malassezia pachydermatis* (67), but was synergistic with cefalexin against methicillin-susceptible and methicillin-resistant *S. pseudintermedius* in a recent study (68).

These data establish local biological activity but do not establish clinical cure rates, optimal contact time, ototoxicity, or compatibility with commercial ear formulations. The need for non-antibiotic adjuncts is strengthened by the broader antimicrobial-resistance context, including documented resistome complexity in veterinary isolates (69). Nevertheless, AMR urgency cannot substitute for clinical validation. Product concentration, pH, ionic strength, biofilm maturity, and concurrent drug class must all be treated as determinants of effect.

The same caution applies to ocular isolates. NAC inhibited common keratitis pathogens *in vitro* (47), but concentrations associated with antimicrobial activity must be reconciled with corneal tolerability and tear dilution. Species-specific *in vitro* systems are valuable for prioritizing interventions, as emphasized by recent canine model literature, but they remain a preclinical layer rather than a direct treatment recommendation. In a retrospective case series of 25 dogs with sialoceles, intracanalicular administration of 10% NAC was associated with a low complication rate, although recurrence and the absence of a comparative control group limit interpretation (70).

A 30-dog comparative study of multicomponent plant-based topical products containing NAC reported improvement in cytology, lesion burden, and pruritus in superficial pyoderma without systemic antimicrobials; because NAC was embedded within complex formulations, its independent contribution cannot be isolated (71). This is a clinically relevant signal, but not evidence for NAC monotherapy.

### 3.9 Neurological and cardiovascular evidence

Two newer experimental domains broaden the translational map without yet changing practice. In a prospective study of nine canine packed-red-cell units stored for 42 days, an NAC–ascorbic-acid additive did not prevent glutathione depletion or outperform the vitamin E–analog regimen for reactive-oxygen-species accumulation (72). In a 15-dog Achilles-tendon defect model, topical NAC improved histological healing relative to untreated controls, but the small sample, limited reporting, and experimental injury design restrict clinical inference (73).

Controlled neurological studies were predominantly negative. In 70 client-owned dogs undergoing hemilaminectomy for acute thoracolumbar intervertebral disc disease, a single preoperative intravenous dose of 50 mg/kg did not improve oxidative biomarkers or neurological recovery (74). A 150 mg/kg dose after global cerebral ischemia and resuscitation similarly failed to improve neurological deficit scores (75). A small protocol combining NAC with other agents in dogs with presumptive degenerative myelopathy did not arrest progression, although the uncontrolled multidrug design prevents attribution (76). These concordant findings do not exclude alternative schedules, but they argue against assuming neuroprotection from antioxidant mechanism alone.

Cardiovascular results were context-dependent. NAC reduced infarct size and reperfusion arrhythmias in one canine coronary occlusion–reperfusion model and reduced infarct size after permanent occlusion in another (77, 78). Other studies found improved regional function or filling pressure without reduced infarct size (79, 80). Protection against rapid-pacing-induced contractile dysfunction was dose dependent, with benefit at 150 mg/kg but not 50 mg/kg (81). In contrast, two independent canine studies found that NAC did not prevent functional doxorubicin cardiotoxicity, although one reported reduced fibrosis (82, 83). The collective pattern is therefore injury- and endpoint-specific rather than a general cardioprotective effect.

### 3.10 Experimental diabetes and multisystem organ protection

A coherent cluster of beagle studies has evaluated NAC combined with insulin in chemically induced type 1 diabetes. Across kidney, liver, brain, myocardium, skeletal muscle, and spleen, the combination attenuated oxidative injury, ferroptosis, mitophagy, pyroptosis, endoplasmic-reticulum stress, apoptosis, and hepatic cholesterol-metabolism abnormalities through pathways including SIRT3–SOD2/GPX4, NLRP3/NF-κB, NRF2/HO-1, and MAPK signaling (84–93).

The consistency is notable, but all reports arose from a closely related experimental platform and research program, rather than from multiple independent bodies of evidence, and focused largely on molecular or histological endpoints. Chemically induced diabetes in purpose-bred beagles does not reproduce the full natural history of spontaneous canine diabetes, and independent replication is absent. A recent Hungarian review of nutritional adjuncts in canine and feline diabetes similarly illustrates the broader attraction of antioxidant supplementation, while emphasizing that mechanistic benefit does not automatically translate into improved glycemic control or complication-free survival (94). The diabetes evidence was therefore classified as experimental despite its volume.

### 3.11 Safety, adverse reactions, and interactions

Oral NAC commonly causes nausea, salivation, or vomiting, in part because of its odor, acidity, and local gastrointestinal effects. Rapid intravenous administration can provoke non-IgE-mediated histamine release with flushing, pruritus, angioedema, hypotension, or bronchospasm. A canine case report documented severe collapse during a first intravenous loading dose, with hypotension, hypothermia, tachycardia, and gallbladder-wall edema requiring epinephrine and supportive treatment (95). Slow diluted infusion and close monitoring are therefore essential, particularly when NAC is used for an indication less compelling than acetaminophen toxicosis.

Species and route matter. Nebulized NAC increased airway resistance in asthmatic cats (9). Topical ophthalmic and otic use exposes tissues to concentrations far above systemic plasma levels, so local tolerability cannot be inferred from intravenous safety. Antimicrobial combinations should be tested rather than assumed to be synergistic (66–68).

The nitrate interaction is nuanced. NAC did not reliably reverse established nitroglycerin tolerance in canine vascular studies (96–98) but could modestly augment vasodilation in non-tolerant preparations and potentiate antiplatelet effects in a coronary-stenosis model (99, 100). Concurrent use may therefore increase hypotensive or antithrombotic effects without restoring responsiveness once tolerance is established. An older adsorption/toxicology study found little *in vitro* adsorption of NAC by activated charcoal; preservation of NAC protection was demonstrated in a mouse acetaminophen model, whereas the canine component evaluated acetaminophen absorption under intravenous NAC protection, so direct inference to oral NAC–charcoal co-administration in dogs remains limited (101). Table 4 summarizes clinically relevant safety considerations.

**Table 4:** Safety, tolerability, and clinically relevant interactions.

Risk or interaction	Evidence	Practical implication
Gastrointestinal intolerance	Vomiting, nausea, salivation, and poor palatability after oral dosing	Give with attention to formulation and aspiration risk; reassess benefit if repeated dosing is poorly tolerated.
IV anaphylactoid/anaphylactic reactions	Histamine-mediated reactions are rate-related; severe first-dose canine collapse has been reported	Dilute, infuse slowly, monitor continuously, and have emergency treatment available.
Feline bronchoconstriction	Nebulized NAC increased airway resistance in experimental feline asthma	Avoid routine inhaled NAC in cats with lower-airway hyper-reactivity.
Topical concentration effects	Corneal healing and antimicrobial effects are concentration-dependent	Do not assume that a higher concentration is more effective or equally tolerable.
Antibiotic/antifungal combinations	Cefalexin synergy contrasted with indifferent or antagonistic effects involving other agents	Evaluate the exact formulation and drug pair; avoid class-wide assumptions.
Nitroglycerin	Does not reliably reverse established tolerance; may augment early vasodilatory or antiplatelet effects	Monitor blood pressure and bleeding risk when co-administered.

### 3.12 Risk of bias and evidence maturity

Risk of bias was lowest in the feline pharmacokinetic crossover study, the hospitalized-dog randomized trial, the intervertebral-disc-disease trial, and the recent feline CKD trial, although each addressed a narrow population or outcome. Several clinical studies lacked blinding, pre-specified primary outcomes, or adequate power. Experimental animal studies frequently omitted allocation concealment, sample-size justification, and blinded outcome assessment. Many older cardiovascular and toxicology studies reported detailed physiological methods but predated current reporting standards.

The *in vitro* otitis and ophthalmic literature generally used defined isolates and quantitative endpoints, but formulation chemistry, pH, vehicle controls, and replicate structure were inconsistent. Confidence was further reduced when multiple mechanistic studies originated from one induced-disease model and research group. Within the predefined evidence-maturity framework, acetaminophen toxicosis represented the most established clinical indication, selected clinical adjuncts were supported or preliminary, and most organ-protective and antimicrobial applications remained experimental or unsupported as tested.

## 4 Discussion

This systematic review identifies a drug with a strong core indication surrounded by a much wider halo of plausible but uneven applications. The principal conclusion is not that NAC is broadly effective because it is an antioxidant. Rather, benefit emerges when a specific pharmacological function matches a defined pathological bottleneck: replenishing thiol substrate during acetaminophen bioactivation, reducing disulfide crosslinks in selected local matrices, or modifying redox injury in a model where timing and exposure are adequate. When that match is absent, neutral and negative results are common.

Acetaminophen toxicosis is qualitatively different from most other indications. In cats, the metabolic vulnerability is species specific, the target pathway is directly engaged, treatment timing has been experimentally examined, and the clinical consequences are immediate. The canine evidence is less extensive but supports accelerated acetaminophen clearance and reversal of oxidative hematological injury. Accordingly, the classic 140 mg/kg loading and 70 mg/kg maintenance approach has a stronger biological and historical basis than dosing schedules used for generic hepatoprotection or systemic inflammation.

The next tier comprises direct but preliminary clinical applications. Two canine parvoviral studies consistently showed improved oxidative and intestinal-injury markers; the feline CKD trial demonstrated clinically familiar renal endpoints; and pilot studies in babesiosis suggested improved hematological and organ-injury profiles. These findings are meaningful because they arose in spontaneous disease, but none yet answers the most important pragmatic questions: whether NAC shortens hospitalization, prevents relapse, improves long-term renal trajectory, or reduces mortality. Replication with concealed allocation, blinded assessment, clinically prioritized endpoints, and formulation-specific dosing is required.

Local antimicrobial use is simultaneously promising and easy to overstate. Milligram-per-milliliter concentrations can inhibit otic and ocular pathogens, and biofilm disruption provides an attractive antibiotic-sparing rationale. Yet the data show that the identity of the companion antimicrobial matters: cefalexin synergy coexists with indifferent or antagonistic interactions involving other classes. A commercial or compounded preparation also changes ionic strength, pH, dwell time, and tissue exposure. The multicomponent pyoderma study provides an initial clinical signal, but it cannot attribute efficacy to NAC. The next step should therefore be controlled clinical trials of chemically defined NAC formulations, not further claims based solely on planktonic MIC values or complex botanical combinations.

Negative findings are central to interpretation. A single perioperative dose did not improve canine spinal-cord outcomes; post-resuscitation treatment did not improve neurological deficit; and repeated NAC did not prevent functional doxorubicin cardiotoxicity. These results indicate that tissue penetration, timing, injury mechanism, and dominant toxic pathway can outweigh redox plausibility. The feline asthma study provides an even stronger warning: local delivery converted a theoretically useful mucolytic into a bronchoconstrictive exposure. Species- and route-specific evidence must therefore supersede generic pharmacological analogy.

The most important knowledge gap is elementary: the pharmacokinetics of conventional aqueous NAC have not been characterized in dogs. This deficiency undermines rational interval selection across hepatobiliary, critical-care, and chronic oral protocols. A modern canine study should quantify reduced, oxidized, and total NAC species; compare oral and intravenous routes; assess food and formulation effects; and link exposure to plasma cysteine, erythrocyte GSH, and tolerability. Population pharmacokinetic sampling in clinical patients could then test whether disease severity, renal function, or body size meaningfully alters exposure.

This review also highlights a publication-structure problem. Ten experimental diabetes reports give the appearance of a large evidence base, but they derive from a closely related induced-disease platform and largely surrogate endpoints. Counting papers without considering model independence would inflate certainty. The same principle applies across veterinary translational research: species-specific cell or isolate systems improve relevance but remain upstream of patient benefit.

The review has limitations. Database-of-origin metadata were unavailable for the legacy reference library, so PRISMA counts could not be reconstructed by individual database. Although all legacy records were re-screened, extracted data and eligibility decisions were subsequently verified, and the update search was documented in full, the absence of preserved database-specific exports for the legacy library and the lack of fully independent duplicate screening throughout reduce auditability relative to a systematic review initiated *de novo* with complete search records and parallel independent assessment. The review was also not prospectively registered. Although the protocol was defined before the update search, the lack of an externally time-stamped protocol limits independent verification that eligibility criteria, outcomes, and synthesis decisions remained unchanged during review conduct and therefore introduces a potential source of review-level bias. The evidence was too heterogeneous for meta-analysis, and design-specific appraisal required judgement across very different study types. Several older reports were available only through abstracts or limited full-text access, and publication bias is likely in experimental organ-protection studies. In addition, restricting eligibility to English- and Hungarian-language full-text publications may have introduced language and selection bias, particularly because some older veterinary NAC literature may have been published in other languages and therefore remained outside the primary evidence synthesis. Finally, the broad scope increases clinical usefulness but reduces the depth possible for any single indication.

These limitations do not alter the main hierarchy. NAC is an established antidote; a plausible and sometimes supported local or systemic adjunct; and an experimental organ-protective agent whose efficacy cannot be generalized. The most productive research agenda is therefore selective: replicate the feline renal signal, test defined topical formulations in otitis and infectious keratitis, resolve canine pharmacokinetics, and use patient-centered endpoints in parvoviral and haemoprotozoan disease.

## 5 Conclusions

N-acetylcysteine has a legitimate but sharply context-dependent place in canine and feline medicine. Early administration for acetaminophen toxicosis is supported by the strongest species-specific evidence. Controlled studies also support further clinical evaluation in hospitalized dogs with defined oxidative depletion, canine parvoviral enteritis, feline acute-on-chronic kidney disease, and selected topical ophthalmic or otic applications. By contrast, many frequently discussed indications remain experimental, and several controlled studies have shown no benefit or route-specific harm. Clinicians should distinguish trial-tested regimens from consensus-derived schedules, avoid extrapolating nebulized canine practice to cats, and treat antimicrobial combinations as formulation- and drug-specific. The field now requires fewer broad claims and more exposure-informed, species-specific trials with clinically meaningful outcomes.

## Data Availability

The original contributions presented in the study are included in the article/Supplementary material, further inquiries can be directed to the corresponding author.
