# Supplementary material for: From antidote to multimodal adjunct: a systematic review of N-acetylcysteine in dogs and cats
Source: Front Vet Sci. 2026 Sep 16;13:1959626. doi: 10.3389/fvets.2026.1959626 (PMC13623545; doi:10.3389/fvets.2026.1959626)
Supplement: Supplementary file 1 [file Supplementary_file_1.docx]

Supplementary Material

# Supplementary Table S1. Search strategies.

| **Source** | **Coverage** | **Search syntax / method** |
| --- | --- | --- |
| PubMed/MEDLINE | Inception–1 Aug 2026 | ("Acetylcysteine"[Mesh] OR "N-acetylcysteine"[tiab] OR acetylcysteine[tiab] OR "N-acetyl-L-cysteine"[tiab] OR NAC[tiab]) AND ("Dogs"[Mesh] OR "Cats"[Mesh] OR dog[tiab] OR dogs[tiab] OR canine[tiab] OR canines[tiab] OR beagle[tiab] OR beagles[tiab] OR cat[tiab] OR cats[tiab] OR feline[tiab] OR felines[tiab] OR "small animal"[tiab] OR "small animals"[tiab] OR "companion animal"[tiab] OR "companion animals"[tiab]) |
| Europe PMC | Inception–1 Aug 2026 | ("N-acetylcysteine" OR acetylcysteine OR "N-acetyl-L-cysteine") AND (dog OR canine OR beagle OR cat OR feline) |
| Crossref and publisher records | Inception–1 Aug 2026 | Exact concept searches combining NAC synonyms with canine/feline terms; DOI resolution and publisher-site verification for candidate reports. |
| Google Scholar | Inception–1 Aug 2026 | All-title and keyword combinations of NAC synonyms with dog/canine/beagle/cat/feline; the first 200 relevance-ranked results were reviewed for the update. |
| MTMT, MATARKA, HuVetA, MÁL and Acta Vet. Hung. archives | Inception–1 Aug 2026 | (N-acetilcisztein OR acetilcisztein OR NAC) AND (kutya OR macska); English equivalents were also applied where supported. |
| Other methods | Through 1 Aug 2026 | Backward reference checking of included studies and relevant reviews; forward citation searching of sentinel pharmacokinetic, acetaminophen, otitis, ophthalmic, and clinical-trial reports. |

# Supplementary Table S2. Characteristics of the 71 primary reports included in the qualitative synthesis.

| **Domain** | **Species** | **Design** | **Study** | **Population / model** | **NAC exposure** | **Main finding** | **Principal limitation** |
| --- | --- | --- | --- | --- | --- | --- | --- |
| Acetaminophen | Cat | Controlled challenge | St. Omer & McKnight, 1980 | Experimental cats | Oral NAC loading + repeated dosing | Protection was dose- and challenge-dependent; untreated animals died | Some regimen details and reporting reflect older standards |
| Acetaminophen | Cat | Controlled challenge | Gaunt et al., 1981 | Experimental cats | Repeated oral NAC | Earlier treatment reduced methaemoglobinaemia and oxidative erythrocyte injury | Small study; induced toxicosis |
| Acetaminophen | Dog | Pharmacokinetic toxicology | St. Omer & Mohammad, 1984 | Beagles | 140 mg/kg then 70 mg/kg oral doses | Reduced acetaminophen half-life and increased clearance | Small purpose-bred cohort |
| Acetaminophen | Dog + cat | Experimental toxicology | Savides et al., 1985 | Dogs and cats | NAC considered in toxicological context | Characterized marked species differences in toxicity and biotransformation | Not an efficacy trial |
| Acetaminophen | Cat | Controlled comparison | Rumbeiha et al., 1995 | Experimental cats | NAC vs methylene blue / combinations | NAC produced the best overall response among tested regimens | Induced model; older reporting |
| Acetaminophen | Cat | Controlled challenge | Avizeh et al., 2010 | Experimental cats | NAC with/without silymarin | Supported hepatoprotection; timing influenced response | Combination arms complicate attribution |
| Acetaminophen | Dog | Case report | MacNaughton, 2003 | 1 dog | IV NAC within multimodal treatment | Resolution of methaemoglobinaemia and haemolysis | Single case; co-interventions |
| PK | Cat | Randomized crossover PK | Buur et al., 2013 | 6 healthy cats | 100 mg/kg PO and IV | Oral bioavailability 19.3%; rapid elimination | Healthy cats; no PD endpoint |
| PK / safety | Dog | Formulation PK/safety | Alipour et al., 2013 | Beagles | Liposomal NAC IV | Formulation disposition and safety characterized | Not conventional aqueous NAC |
| Critical illness | Dog | Randomized blinded clinical trial | Viviano & VanderWielen, 2013 | 60 ill dogs + 14 healthy controls | NAC during first 48 h | Increased cysteine / maintained GSH; no survival benefit | Heterogeneous diagnoses |
| Critical illness | Dog | Experimental endotoxic shock | Bakker et al., 1994 | Experimental dogs | Loading + maintenance infusion | Improved oxygen utilization, lactate clearance and TNF response | Induced model |
| Hyperoxic lung injury | Dog | Experimental ventilation | Wagner et al., 1989 | Experimental dogs | Continuous IV infusion | Delayed pulmonary vascular and compliance injury | Extreme high-FiO2 model |
| Cardiopulmonary bypass | Dog | Experimental surgery | Qu et al., 2013 | Experimental dogs | Perioperative NAC | Reduced histological lung injury and oxidative indices | Short-term induced model |
| Haemodilution | Dog | Experimental physiology | Kerbaul et al., 2004 | Experimental dogs | 200 mg/kg IV pretreatment | Preserved hypoxic pulmonary vasoconstriction | High-dose anaesthetized model |
| Respiratory | Dog | Clinical case series | Staudacher & Staudacher, 1989 | Dogs with chronic bronchitis | Acetylcysteine-containing protocol | Improved dyspnoea and gas-exchange observations | Small, historical, multimodal |
| Respiratory | Dog | Experimental bronchoprovocation | Ueno et al., 1989 | Experimental dogs | NAC around methacholine/isoprenaline challenge | Context-dependent gas-exchange effects | Not spontaneous disease |
| Respiratory | Dog | Ex vivo physiology | Yanaura et al., 1981 | Canine tracheal cilia | Range of concentrations | Low exposure stimulated, high exposure depressed ciliary activity | Ex vivo model |
| Respiratory | Cat | Controlled asthma model | Reinero et al., 2011 | Cats with experimental asthma | Endotracheal nebulization | Increased airway resistance | Induced model but direct safety relevance |
| Cornea | Dog | Controlled wound model | Aldavood et al., 2003 | Experimental canine corneal wounds | 3%, 10%, or 20% topical NAC | 3% accelerated healing; higher concentrations did not | Non-infected wound model |
| Cornea | Dog | Ex vivo tear-film assay | Couture et al., 2006 | Normal canine tear film | 10% topical NAC | Limited gelatinase inhibition | Normal eyes; surrogate endpoint |
| Cornea | Dog | In vitro degradation model | Kimmitt et al., 2018 | Canine corneal tissue model | Multiple concentrations/combinations | Concentration-dependent collagenase inhibition | In vitro; comparator-dependent |
| Keratitis | Dog + cat | In vitro isolate study | Walter et al., 2023 | 37 clinical isolates + control strain | NAC concentration series | Inhibited common infectious-keratitis pathogens | No clinical efficacy or tolerance testing |
| Bile | Dog | Ex vivo viscosity study | Boullhesen-Williams et al., 2019 | Normal canine bile | 20% NAC diluted 1:4 | Reduced viscosity, similar to water dilution | Normal bile; dilution confounding |
| Obstructive jaundice | Dog | Experimental surgery | Kigawa et al., 2000 | Experimental dogs | IV NAC | Improved microcirculation, hepatic GSH and ATP | Induced obstruction |
| Hepatic I/R | Dog | Experimental surgery | Baumann et al., 2008 | Experimental dogs | 150 mg/kg IV | No short-term protection of functional/biochemical endpoints | Short reperfusion interval |
| Parvovirus | Dog | Controlled clinical trial | Gaykwad et al., 2018 | CPV-positive puppies | 70 mg/kg IV daily for 5 d | Improved leukocyte recovery and oxidative markers | Small, non-blinded |
| Parvovirus | Dog | Randomized clinical trial | Chethan et al., 2023 | Outpatient CPV cases | NAC adjunct vs other antioxidants / standard care | Reduced oxidative and intestinal-injury markers | Multiple comparator arms; surrogate outcomes |
| Babesiosis | Dog | Pilot clinical study | Mundassery et al., 2025 | Dogs with *B. gibsoni* | 70 mg/kg PO daily for 5 d | Improved haematological and antioxidant indices | Small; many comparisons non-significant |
| Babesiosis | Dog | Randomized pilot clinical study | Khan et al., 2026 | 24 dogs with *B. gibsoni* | 70 mg/kg PO daily for 14 d | Earlier improvement in anaemia, acute phase and hepatic markers | Pilot; not powered for relapse/survival |
| Pancytopenia | Dog | Translational + clinical study | Yadav et al., 2025 | Rat model + clinical canine cases | 50 mg/kg in rat model; canine adjunct protocol | Favourable marrow and haematological changes | Cross-species extrapolation and limited clinical controls |
| CKD | Cat | Double-blind placebo-controlled trial | Alihosseini et al., 2026 | 50 cats with acute-on-chronic CKD | 70 mg/kg IV daily for 7 d | Greater improvement in creatinine, SDMA, BUN and UPC | Single centre; short-term course |
| Renal I/R | Dog | Experimental vascular occlusion | Salom et al., 1998 | Experimental dogs | Intra-arterial NAC pretreatment | Improved GFR recovery and endothelial response | Induced model |
| Renal transplantation | Dog | Experimental transplantation | Lin et al., 2004 | Autologous canine grafts | NAC + AICAR combination | Markedly improved graft function | NAC contribution not separable |
| Otitis | Dog | In vitro isolate study | May et al., 2016 | Canine otitis isolates | NAC concentration series | Direct antibacterial activity | No biofilm or clinical endpoint |
| Otitis | Dog | In vitro isolate study | Chan et al., 2019 | Otic pathogens + type strains | Seven adjuvants including NAC | NAC active against major otic pathogens | Formulation not clinical |
| Otitis biofilm | Dog | In vitro biofilm study | Chan et al., 2019 | Canine otitis isolates | MBEC testing | Established biofilms required high concentrations | No tissue-tolerance assessment |
| Otitis combinations | Dog | In vitro interaction study | May et al., 2019 | Canine bacterial isolates | NAC + enrofloxacin/gentamicin | Mostly indifferent or antagonistic interactions | Limited drug classes |
| Otitis yeast | Dog | In vitro interaction study | Jeon & Bae, 2024 | 13 *M. pachydermatis* isolates | NAC ± antifungals | Direct inhibition; little consistent synergy | Small isolate panel |
| Otitis staphylococci | Dog | In vitro interaction study | Hawwash et al., 2026 | MSSP and MRSP isolates | NAC ± cefalexin | Rapid bactericidal effect and cefalexin synergy | In vitro; clinical concentrations untested |
| Sialocele | Dog | Retrospective case series | Ortillés et al., 2020 | 25 dogs | 10% intracanalicular NAC | Low complication rate; recurrences medically controlled | No comparator; incomplete follow-up |
| Spinal cord injury | Dog | Randomized blinded clinical trial | Baltzer et al., 2008 | 70 dogs with acute IVDD | 50 mg/kg IV preoperative | No biomarker or neurological benefit | Single dose |
| Global cerebral ischaemia | Dog | Experimental resuscitation | Silbergleit et al., 1999 | Experimental dogs | 150 mg/kg IV post-resuscitation | No neurological benefit | Short follow-up |
| Degenerative myelopathy | Dog | Uncontrolled clinical protocol | Polizopoulou et al., 2008 | 12 dogs | NAC within multidrug protocol | Progressive deterioration | Tentative diagnosis; no control |
| Myocardial I/R | Dog | Experimental occlusion/reperfusion | Šochman et al., 1990 | Experimental dogs | 100 mg/kg bolus + infusion | Reduced infarct size and reperfusion arrhythmias | Short reperfusion model |
| Permanent coronary occlusion | Dog | Experimental occlusion | Alberola et al., 1991 | Experimental dogs | 150 mg/kg IV | Reduced infarct size and preserved GSH | Permanent occlusion model |
| Myocardial I/R | Dog | Experimental occlusion/reperfusion | Forman et al., 1988 | Experimental dogs | NAC during ischaemia/reperfusion | Improved wall motion; no infarct-size reduction | Mixed endpoints |
| Myocardial I/R | Dog | Experimental occlusion/reperfusion | Tripathi & Hegde, 1998 | Experimental dogs | NAC at reperfusion | No infarct-size reduction; filling pressure improved | Limited reporting |
| Rapid pacing | Dog | Experimental pacing | Gare et al., 2002 | Experimental dogs | 50 or 150 mg/kg pretreatment | Protection only at 150 mg/kg | Acute model |
| Doxorubicin cardiotoxicity | Dog | Placebo-controlled chronic study | Unverferth et al., 1985 | Dogs receiving doxorubicin | Low- and high-dose NAC protocols | No functional or survival protection; less fibrosis at high dose | Older oncology model |
| Doxorubicin cardiotoxicity | Dog | Comparative experimental study | Herman et al., 1985 | Beagles | 200 mg/kg before doxorubicin | No reduction in myocardial lesions | Compared with dexrazoxane-like agent |
| Nitroglycerin tolerance | Dog | Experimental vascular study | Abdollah et al., 1987 | Canine vascular rings | NAC + nitroglycerin | Did not modify established tolerance | In vitro vascular model |
| Nitroglycerin tolerance | Dog | Chronic instrumentation | Münzel et al., 1989 | Instrumented dogs | NAC during nitrate exposure | No reversal of tolerance; modest tolerance-independent interaction | Complex vascular endpoints |
| Nitroglycerin tolerance | Dog | Chronic infusion | Holtz et al., 1989 | Instrumented dogs | NAC during long-term nitrate infusion | No reliable reversal of tolerance | Related research platform |
| Nitroglycerin interaction | Dog | Isolated coronary arteries | Ohba et al., 1987 | Canine coronary arteries | NAC / nitrate comparison | Tendency to potentiate relaxation and reduce acute tolerance | Ex vivo |
| Platelet thrombosis | Dog | Coronary stenosis model | Folts et al., 1991 | Experimental dogs | NAC + nitroglycerin | Potentiated inhibition of cyclic platelet thrombosis | Experimental antithrombotic endpoint |
| Activated charcoal | Dog + other | Adsorption / toxicology study | Van de Graaff et al., 1982 | In vitro + canine AP absorption + mouse lethality | Charcoal with NAC context | Little NAC adsorption; protection retained in mouse model | Canine NAC concentrations not measured |
| T1DM kidney | Dog | Experimental diabetes | Li et al., 2022 | Beagles with induced T1DM | Insulin ± NAC | Reduced ferroptosis via SIRT3–SOD2/GPX4 | Single research platform |
| T1DM kidney | Dog | Experimental diabetes | Ma et al., 2023 | Beagles with induced T1DM | Insulin ± NAC | Improved mitochondrial dynamics / mitophagy | Surrogate endpoints |
| T1DM liver | Dog | Experimental diabetes | Huo et al., 2022 | Beagles with induced T1DM | Insulin ± NAC | Reduced NLRP3/NF-κB-related pyroptosis | Surrogate endpoints |
| T1DM brain | Dog | Experimental diabetes | Li et al., 2022 | Beagles with induced T1DM | Insulin ± NAC | Reduced oxidative injury and preserved BBB proteins | Surrogate endpoints |
| T1DM myocardium | Dog | Experimental diabetes | Huang et al., 2023 | Beagles with induced T1DM | Insulin ± NAC | Reduced TNF-mediated apoptosis | Surrogate endpoints |
| T1DM muscle | Dog | Experimental diabetes | Ding et al., 2023 | Beagles with induced T1DM | Insulin ± NAC | Reduced atrophy via NRF2/HO-1 | Surrogate endpoints |
| T1DM myocardium | Dog | Experimental diabetes | Wu et al., 2024 | Beagles with induced T1DM | Insulin ± NAC | Reduced ER-stress-related myocardial injury | Surrogate endpoints |
| T1DM spleen | Dog | Experimental diabetes | Zhang et al., 2024 | Beagles with induced T1DM | Insulin ± NAC | Reduced MAPK/NF-κB signalling and pyroptosis | Surrogate endpoints |
| T1DM liver transcriptomics | Dog | Experimental diabetes | Zhao et al., 2025 | Beagle T1DM model | NAC-related transcriptomic comparison | CXCL12 identified as candidate hub | Exploratory omics; no clinical endpoint |
| Anaphylaxis | Dog | Case report | Gregory & Binagia, 2022 | 1 dog | First IV loading dose | Severe immediate reaction requiring epinephrine | Single case; mechanism uncertain |
| T1DM cholesterol metabolism | Dog | Randomized experimental diabetes | Wang et al., 2023 | 40 beagles in five groups | Insulin ± NAC for 120 d | Improved hepatic cholesterol-metabolism and injury indices | Induced model; same research platform |
| Achilles tendon repair | Dog | Controlled experimental injury | Nishemi et al., 2024 | 15 adult dogs | Topical NAC vs PRP vs untreated control | Improved histological tendon healing at 42 d | Small sample and limited reporting |
| Superficial pyoderma | Dog | Prospective comparative clinical study | Waisburd et al., 2025 | 30 dogs | Multicomponent NAC-containing topicals vs medicated shampoo | Improved cytology, lesion and pruritus scores | NAC effect not separable from co-ingredients |
| Infectious keratitis | Dog + cat | In vitro isolate study | Wolff et al., 2025 | 27 clinical isolates plus controls | Commercial antiseptics including NAC | Bactericidal activity; strong matrix effect | Predominantly canine isolates; no in vivo validation |
| Packed red-cell storage | Dog | Prospective ex vivo comparative study | Roque-Torres et al., 2025 | 9 canine pRBC units | NAC + ascorbic acid vs vitamin E analog vs saline | NAC combination did not prevent GSH depletion | Combination exposure; storage model |

# Supplementary Table S3. Design-specific risk-of-bias summary.

| **Study** | **Species** | **Design** | **Overall appraisal** | **Main basis** |
| --- | --- | --- | --- | --- |
| St. Omer & McKnight, 1980 | Cat | Controlled challenge | Some concerns to high | Induced models were generally well described, but randomization, blinding, and sample-size justification were often absent. |
| Gaunt et al., 1981 | Cat | Controlled challenge | Some concerns to high | Induced models were generally well described, but randomization, blinding, and sample-size justification were often absent. |
| St. Omer & Mohammad, 1984 | Dog | Pharmacokinetic toxicology | Some concerns | Clinical relevance was direct, but design or reporting did not fully address confounding and selective outcome reporting. |
| Savides et al., 1984 | Dog + cat | Experimental toxicology | Some concerns to high | Induced models were generally well described, but randomization, blinding, and sample-size justification were often absent. |
| Rumbeiha et al., 1995 | Cat | Controlled comparison | Some concerns | Clinical relevance was direct, but design or reporting did not fully address confounding and selective outcome reporting. |
| Avizeh et al., 2010 | Cat | Controlled challenge | Some concerns to high | Induced models were generally well described, but randomization, blinding, and sample-size justification were often absent. |
| MacNaughton, 2003 | Dog | Case report | High | No concurrent comparator and substantial susceptibility to selection, confounding, or outcome-assessment bias. |
| Buur et al., 2013 | Cat | Randomized crossover PK | Low to some concerns | Randomization or blinding reported; uncertainty commonly remained for allocation concealment, prespecification, or power. |
| Alipour et al., 2013 | Dog | Formulation PK/safety | Some concerns | Clinical relevance was direct, but design or reporting did not fully address confounding and selective outcome reporting. |
| Viviano & VanderWielen, 2013 | Dog | Randomized blinded clinical trial | Low to some concerns | Randomization or blinding reported; uncertainty commonly remained for allocation concealment, prespecification, or power. |
| Bakker et al., 1994 | Dog | Experimental endotoxic shock | Some concerns to high | Induced models were generally well described, but randomization, blinding, and sample-size justification were often absent. |
| Wagner et al., 1989 | Dog | Experimental ventilation | Some concerns to high | Induced models were generally well described, but randomization, blinding, and sample-size justification were often absent. |
| Qu et al., 2013 | Dog | Experimental surgery | Some concerns to high | Induced models were generally well described, but randomization, blinding, and sample-size justification were often absent. |
| Kerbaul et al., 2004 | Dog | Experimental physiology | Some concerns to high | Induced models were generally well described, but randomization, blinding, and sample-size justification were often absent. |
| Staudacher & Staudacher, 1989 | Dog | Clinical case series | High | No concurrent comparator and substantial susceptibility to selection, confounding, or outcome-assessment bias. |
| Ueno et al., 1989 | Dog | Experimental bronchoprovocation | Some concerns to high | Induced models were generally well described, but randomization, blinding, and sample-size justification were often absent. |
| Yanaura et al., 1981 | Dog | Ex vivo physiology | Moderate to high | Quantitative endpoints were common, but replicate structure, formulation chemistry, vehicle controls, or clinical relevance were variably reported. |
| Reinero et al., 2011 | Cat | Controlled asthma model | Some concerns | Clinical relevance was direct, but design or reporting did not fully address confounding and selective outcome reporting. |
| Aldavood et al., 2003 | Dog | Controlled wound model | Some concerns | Clinical relevance was direct, but design or reporting did not fully address confounding and selective outcome reporting. |
| Couture et al., 2006 | Dog | Ex vivo tear-film assay | Moderate to high | Quantitative endpoints were common, but replicate structure, formulation chemistry, vehicle controls, or clinical relevance were variably reported. |
| Kimmitt et al., 2018 | Dog | In vitro degradation model | Moderate to high | Quantitative endpoints were common, but replicate structure, formulation chemistry, vehicle controls, or clinical relevance were variably reported. |
| Walter et al., 2023 | Dog + cat | In vitro isolate study | Moderate to high | Quantitative endpoints were common, but replicate structure, formulation chemistry, vehicle controls, or clinical relevance were variably reported. |
| Boullhesen-Williams et al., 2019 | Dog | Ex vivo viscosity study | Moderate to high | Quantitative endpoints were common, but replicate structure, formulation chemistry, vehicle controls, or clinical relevance were variably reported. |
| Kigawa et al., 2000 | Dog | Experimental surgery | Some concerns to high | Induced models were generally well described, but randomization, blinding, and sample-size justification were often absent. |
| Baumann et al., 2008 | Dog | Experimental surgery | Some concerns to high | Induced models were generally well described, but randomization, blinding, and sample-size justification were often absent. |
| Gaykwad et al., 2018 | Dog | Controlled clinical trial | Some concerns | Clinical relevance was direct, but design or reporting did not fully address confounding and selective outcome reporting. |
| Chethan et al., 2023 | Dog | Randomized clinical trial | Low to some concerns | Randomization or blinding reported; uncertainty commonly remained for allocation concealment, prespecification, or power. |
| Mundassery et al., 2025 | Dog | Pilot clinical study | Some concerns | Clinical relevance was direct, but design or reporting did not fully address confounding and selective outcome reporting. |
| Khan et al., 2026 | Dog | Randomized pilot clinical study | Low to some concerns | Randomization or blinding reported; uncertainty commonly remained for allocation concealment, prespecification, or power. |
| Yadav et al., 2025 | Dog | Translational + clinical study | Some concerns | Clinical relevance was direct, but design or reporting did not fully address confounding and selective outcome reporting. |
| Alihosseini et al., 2026 | Cat | Double-blind placebo-controlled trial | Low to some concerns | Randomization or blinding reported; uncertainty commonly remained for allocation concealment, prespecification, or power. |
| Salom et al., 1998 | Dog | Experimental vascular occlusion | Some concerns to high | Induced models were generally well described, but randomization, blinding, and sample-size justification were often absent. |
| Lin et al., 2004 | Dog | Experimental transplantation | Some concerns to high | Induced models were generally well described, but randomization, blinding, and sample-size justification were often absent. |
| May et al., 2016 | Dog | In vitro isolate study | Moderate to high | Quantitative endpoints were common, but replicate structure, formulation chemistry, vehicle controls, or clinical relevance were variably reported. |
| Chan et al., 2019 | Dog | In vitro isolate study | Moderate to high | Quantitative endpoints were common, but replicate structure, formulation chemistry, vehicle controls, or clinical relevance were variably reported. |
| Chan et al., 2019 | Dog | In vitro biofilm study | Moderate to high | Quantitative endpoints were common, but replicate structure, formulation chemistry, vehicle controls, or clinical relevance were variably reported. |
| May et al., 2019 | Dog | In vitro interaction study | Moderate to high | Quantitative endpoints were common, but replicate structure, formulation chemistry, vehicle controls, or clinical relevance were variably reported. |
| Jeon & Bae, 2024 | Dog | In vitro interaction study | Moderate to high | Quantitative endpoints were common, but replicate structure, formulation chemistry, vehicle controls, or clinical relevance were variably reported. |
| Hawwash et al., 2026 | Dog | In vitro interaction study | Moderate to high | Quantitative endpoints were common, but replicate structure, formulation chemistry, vehicle controls, or clinical relevance were variably reported. |
| Ortillés et al., 2020 | Dog | Retrospective case series | High | No concurrent comparator and substantial susceptibility to selection, confounding, or outcome-assessment bias. |
| Baltzer et al., 2008 | Dog | Randomized blinded clinical trial | Low to some concerns | Randomization or blinding reported; uncertainty commonly remained for allocation concealment, prespecification, or power. |
| Silbergleit et al., 1999 | Dog | Experimental resuscitation | Some concerns to high | Induced models were generally well described, but randomization, blinding, and sample-size justification were often absent. |
| Polizopoulou et al., 2008 | Dog | Uncontrolled clinical protocol | High | No concurrent comparator and substantial susceptibility to selection, confounding, or outcome-assessment bias. |
| Šochman et al., 1990 | Dog | Experimental occlusion/reperfusion | Some concerns to high | Induced models were generally well described, but randomization, blinding, and sample-size justification were often absent. |
| Alberola et al., 1991 | Dog | Experimental occlusion | Some concerns to high | Induced models were generally well described, but randomization, blinding, and sample-size justification were often absent. |
| Forman et al., 1988 | Dog | Experimental occlusion/reperfusion | Some concerns to high | Induced models were generally well described, but randomization, blinding, and sample-size justification were often absent. |
| Tripathi & Hegde, 1998 | Dog | Experimental occlusion/reperfusion | Some concerns to high | Induced models were generally well described, but randomization, blinding, and sample-size justification were often absent. |
| Gare et al., 2002 | Dog | Experimental pacing | Some concerns to high | Induced models were generally well described, but randomization, blinding, and sample-size justification were often absent. |
| Unverferth et al., 1985 | Dog | Placebo-controlled chronic study | Some concerns | Clinical relevance was direct, but design or reporting did not fully address confounding and selective outcome reporting. |
| Herman et al., 1985 | Dog | Comparative experimental study | Some concerns to high | Induced models were generally well described, but randomization, blinding, and sample-size justification were often absent. |
| Abdollah et al., 1987 | Dog | Experimental vascular study | Some concerns to high | Induced models were generally well described, but randomization, blinding, and sample-size justification were often absent. |
| Münzel et al., 1989 | Dog | Chronic instrumentation | Some concerns | Clinical relevance was direct, but design or reporting did not fully address confounding and selective outcome reporting. |
| Holtz et al., 1989 | Dog | Chronic infusion | Some concerns | Clinical relevance was direct, but design or reporting did not fully address confounding and selective outcome reporting. |
| Ohba et al., 1987 | Dog | Isolated coronary arteries | Moderate to high | Quantitative endpoints were common, but replicate structure, formulation chemistry, vehicle controls, or clinical relevance were variably reported. |
| Folts et al., 1991 | Dog | Coronary stenosis model | Some concerns | Clinical relevance was direct, but design or reporting did not fully address confounding and selective outcome reporting. |
| Van de Graaff et al., 1982 | Dog + other | Adsorption / toxicology study | Some concerns | Clinical relevance was direct, but design or reporting did not fully address confounding and selective outcome reporting. |
| Li et al., 2022 | Dog | Experimental diabetes | Some concerns to high | Induced models were generally well described, but randomization, blinding, and sample-size justification were often absent. |
| Ma et al., 2023 | Dog | Experimental diabetes | Some concerns to high | Induced models were generally well described, but randomization, blinding, and sample-size justification were often absent. |
| Huo et al., 2022 | Dog | Experimental diabetes | Some concerns to high | Induced models were generally well described, but randomization, blinding, and sample-size justification were often absent. |
| Li et al., 2022 | Dog | Experimental diabetes | Some concerns to high | Induced models were generally well described, but randomization, blinding, and sample-size justification were often absent. |
| Huang et al., 2023 | Dog | Experimental diabetes | Some concerns to high | Induced models were generally well described, but randomization, blinding, and sample-size justification were often absent. |
| Ding et al., 2023 | Dog | Experimental diabetes | Some concerns to high | Induced models were generally well described, but randomization, blinding, and sample-size justification were often absent. |
| Wu et al., 2024 | Dog | Experimental diabetes | Some concerns to high | Induced models were generally well described, but randomization, blinding, and sample-size justification were often absent. |
| Zhang et al., 2024 | Dog | Experimental diabetes | Some concerns to high | Induced models were generally well described, but randomization, blinding, and sample-size justification were often absent. |
| Zhao et al., 2025 | Dog | Experimental diabetes | Some concerns to high | Induced models were generally well described, but randomization, blinding, and sample-size justification were often absent. |
| Gregory & Binagia, 2022 | Dog | Case report | High | No concurrent comparator and substantial susceptibility to selection, confounding, or outcome-assessment bias. |
| Wang et al., 2023 | Dog | Randomized experimental diabetes | Low to some concerns | Randomization or blinding reported; uncertainty commonly remained for allocation concealment, prespecification, or power. |
| Nishemi et al., 2024 | Dog | Controlled experimental injury | Some concerns to high | Induced models were generally well described, but randomization, blinding, and sample-size justification were often absent. |
| Waisburd et al., 2025 | Dog | Prospective comparative clinical study | Some concerns | Clinical relevance was direct, but design or reporting did not fully address confounding and selective outcome reporting. |
| Wolff et al., 2025 | Dog + cat | In vitro isolate study | Moderate to high | Quantitative endpoints were common, but replicate structure, formulation chemistry, vehicle controls, or clinical relevance were variably reported. |
| Roque-Torres et al., 2025 | Dog | Prospective ex vivo comparative study | Moderate to high | Quantitative endpoints were common, but replicate structure, formulation chemistry, vehicle controls, or clinical relevance were variably reported. |

**Supplementary Table S4.** Full-text reports excluded from the primary-study qualitative synthesis and primary reason for exclusion. The table lists the 45 records from the 104-record topic-curated legacy evidence library that did not meet the prespecified eligibility criteria. One primary exclusion reason was assigned to each report. The 12 additional NAC-specific reports identified during the update search met the eligibility criteria and are represented among the included studies in Supplementary Tables S2 and S3. Exclusion categories comprised secondary/background sources without original eligible NAC data (n = 26), studies with no direct NAC exposure (n = 13), and studies without an eligible canine/feline model (n = 6).

| **No.** | **Full citation** | **Primary exclusion reason** | **Rationale** |
| --- | --- | --- | --- |
| 1 | Aldini G, Altomare A, Baron G, Vistoli G, Carini M, Borsani L, Sergio F (2018) N-Acetylcysteine as an antioxidant and disulphide-breaking agent: the reasons why. Free Radical Research 52(7):751-762. https://doi.org/10.1080/10715762.2018.1468564 | Secondary/background source without original eligible NAC data | Narrative mechanistic review; no original canine/feline NAC dataset. |
| 2 | Aruoma OI, Halliwell B, Hoey BM, Butler J (1989) The antioxidant action of N-acetylcysteine: its reaction with hydrogen peroxide, hydroxyl radical, superoxide, and hypochlorous acid. Free Radical Biology and Medicine 6(6):593-597. https://doi.org/10.1016/0891-5849(89)90066-x | No eligible canine/feline model | Cell-free chemical reaction study; no canine- or feline-derived model. |
| 3 | Atkuri KR, Mantovani JJ, Herzenberg LA, Herzenberg LA (2007) N-Acetylcysteine—a safe antidote for cysteine/glutathione deficiency. Current Opinion in Pharmacology 7(4):355-359. https://doi.org/10.1016/j.coph.2007.04.005 | Secondary/background source without original eligible NAC data | Narrative pharmacological review; no original eligible veterinary NAC data. |
| 4 | Barnes PJ, Karin M (1997) Nuclear factor-κB: a pivotal transcription factor in chronic inflammatory diseases. New England Journal of Medicine 336(15):1066-1071. https://doi.org/10.1056/NEJM199704103361506 | Secondary/background source without original eligible NAC data | General review of NF-kappaB biology; no original NAC intervention dataset. |
| 5 | Blanca PM, María Luisa FR, Guadalupe M, Fátima CL (2024) Oxidative Stress in Canine Diseases: A Comprehensive Review. Antioxidants (Basel, Switzerland), 13(11):1396. https://doi.org/10.3390/antiox13111396 | Secondary/background source without original eligible NAC data | Review of oxidative stress in canine diseases; no original NAC intervention data. |
| 6 | Borgström L, Kågedal B, Paulsen O (1986) Pharmacokinetics of N-acetylcysteine in man. European Journal of Clinical Pharmacology 31(2):217-222. https://doi.org/10.1007/BF00606662 | No eligible canine/feline model | Human pharmacokinetic study; no canine or feline participants or material. |
| 7 | Brooks DE, Ollivier FJ (2004) Matrix metalloproteinase inhibition in corneal ulceration. Veterinary Clinics of North America: Small Animal Practice 34(3):611-622. https://doi.org/10.1016/j.cvsm.2003.12.005 | Secondary/background source without original eligible NAC data | Clinical review of corneal collagenolysis; no original NAC experiment. |
| 8 | Center SA, Warner KL, Erb HN (2002) Liver glutathione concentrations in dogs and cats with naturally occurring liver disease. American Journal of Veterinary Research 63(8):1187-1197. https://doi.org/10.2460/ajvr.2002.63.1187 | No direct NAC exposure | Measured hepatic glutathione in dogs and cats but did not administer or test NAC. |
| 9 | Chen H, Segev G (2024) Evaluation of oxidative stress in dogs and cats with chronic kidney disease. Journal of veterinary internal medicine, 38(6):3105–3110. https://doi.org/10.1111/jvim.17230 | No direct NAC exposure | Evaluated oxidative stress in canine/feline CKD without direct NAC exposure. |
| 10 | Cotgreave IA (1997) N-acetylcysteine: pharmacological considerations and experimental and clinical applications. Advances in Pharmacology 38:205-227. | Secondary/background source without original eligible NAC data | Broad pharmacological review; no original eligible canine/feline NAC dataset. |
| 11 | Court MH, Greenblatt DJ (1997) Molecular basis for deficient acetaminophen glucuronidation in cats. Biochemical Pharmacology 53(7):1041-1047. https://doi.org/10.1016/s0006-2952(97)00072-5 | No direct NAC exposure | Feline acetaminophen glucuronidation study without NAC administration. |
| 12 | Dekhuijzen PNR (2004) Antioxidant properties of N-acetylcysteine: their relevance in relation to chronic obstructive pulmonary disease. European Respiratory Journal 23(4):629-636. https://doi.org/10.1183/09031936.04.00016804 | Secondary/background source without original eligible NAC data | Human respiratory review; no original canine/feline NAC dataset. |
| 13 | Denzoin Vulcano LA, Confalonieri O, Franci R, Tapia MO, Soraci AL (2013) Efficacy of free glutathione and niosomal glutathione in the treatment of acetaminophen-induced hepatotoxicity in cats. Open Veterinary Journal 3(1):56-63. | No direct NAC exposure | Compared free and niosomal glutathione in cats; NAC was not evaluated. |
| 14 | Freeman LM, Brown DJ, Rush JE (1999) Assessment of degree of oxidative stress and antioxidant concentrations in dogs with idiopathic dilated cardiomyopathy. Journal of the American Veterinary Medical Association 215(5):644-646. | No direct NAC exposure | Characterized oxidative stress in canine cardiomyopathy without NAC exposure. |
| 15 | Ishikawa S, Takemitsu H, Li G, Mori N, Yamamoto I, Arai T (2015) Short communication: molecular characterization of dog and cat p65 subunits of NF-κB. Research in Veterinary Science 99:145-148. https://doi.org/10.1016/j.rvsc.2015.02.009 | No direct NAC exposure | Molecular characterization of canine/feline NF-kappaB p65 without NAC treatment. |
| 16 | Kanyorszky ET, Sterczer Á, Nabulsi S, Manczur F (2024) Akut hasnyálmirigy-gyulladás kutyákban és emberekben: Irodalmi összefoglaló [Acute pancreatitis in dogs and humans: Literature review]. Magyar Állatorvosok Lapja 146(1):3-22. doi:10.56385/magyallorv.2024.01.3-22 | Secondary/background source without original eligible NAC data | Literature review of acute pancreatitis; no original NAC dataset. |
| 17 | Kapun AP, Salobir J, Levart A, Kotnik T, Svete AN (2012) Oxidative stress markers in canine atopic dermatitis. Research in veterinary science, 92(3):469–470. https://doi.org/10.1016/j.rvsc.2011.04.014 | No direct NAC exposure | Measured oxidative-stress markers in canine atopic dermatitis without NAC exposure. |
| 18 | Liu H, Jiao Y, Wang PC, Chen Y, Xu M, Zhang X, Zheng X, Yang Z (2026) Oxidative stress and antioxidant therapeutic mechanisms. Pharmacology & therapeutics, 278:108962. https://doi.org/10.1016/j.pharmthera.2025.108962 | Secondary/background source without original eligible NAC data | General review of oxidative-stress mechanisms; no original eligible NAC dataset. |
| 19 | Lu SC (2009) Regulation of glutathione synthesis. Molecular Aspects of Medicine 30(1-2):42-59. https://doi.org/10.1016/j.mam.2008.05.005 | Secondary/background source without original eligible NAC data | Mechanistic review of glutathione synthesis; no original canine/feline NAC data. |
| 20 | McMichael MA (2007) Oxidative stress, antioxidants, and assessment of oxidative stress in dogs and cats. Journal of the American Veterinary Medical Association, 231(5):714–720. https://doi.org/10.2460/javma.231.5.714 | Secondary/background source without original eligible NAC data | Veterinary review of oxidative stress and antioxidants; no original NAC dataset. |
| 21 | Nur Mahendra MY, Pertiwi H, Dadi TB, Sofyan MS, Kamaludeen J (2026) N-Acetylcysteine in dogs and cats: pharmacokinetics, therapeutic effects and antioxidant function. Croatian Veterinary Journal 57(2):154-161. https://doi.org/10.46419/cvj.57.2.6 | Secondary/background source without original eligible NAC data | Narrative review of NAC in dogs and cats; secondary publication without new data. |
| 22 | Olsson B, Johansson M, Gabrielsson J, Bolme P (1988) Pharmacokinetics and bioavailability of reduced and oxidized N-acetylcysteine. European Journal of Clinical Pharmacology 34(1):77-82. https://doi.org/10.1007/BF01061422 | No eligible canine/feline model | Human pharmacokinetic/bioavailability study; no canine/feline model. |
| 23 | Rogers DF (2007) Mucoactive agents for airway mucus hypersecretory diseases. Respiratory Care 52(9):1176-1193. | Secondary/background source without original eligible NAC data | Review of mucoactive agents; no original eligible canine/feline NAC dataset. |
| 24 | Rogliani P, Manzetti GM, Gholamalishahi S, Cazzola M, Calzetta L (2024) Impact of N-acetylcysteine on mucus hypersecretion in the airways: a systematic review. International Journal of Chronic Obstructive Pulmonary Disease 19:2347-2360. https://doi.org/10.2147/COPD.S474512 | Secondary/background source without original eligible NAC data | Systematic review of airway mucus hypersecretion; no original canine/feline NAC data. |
| 25 | Rubio CP, Hernández-Ruiz J, Martinez-Subiela S, Tvarijonaviciute A, Ceron JJ (2016) Spectrophotometric assays for total antioxidant capacity (TAC) in dog serum: an update. BMC Veterinary Research 12(1):166. https://doi.org/10.1186/s12917-016-0792-7 | No direct NAC exposure | Analytical study of canine serum antioxidant capacity; NAC was not evaluated. |
| 26 | Rushworth GF, Megson IL (2014) Existing and potential therapeutic uses for N-acetylcysteine: the need for conversion to intracellular glutathione for antioxidant benefits. Pharmacology & Therapeutics 141(2):150-159. https://doi.org/10.1016/j.pharmthera.2013.09.006 | Secondary/background source without original eligible NAC data | Narrative review of therapeutic NAC use; no original eligible veterinary dataset. |
| 27 | Samuni Y, Goldstein S, Dean OM, Berk M (2013) The chemistry and biological activities of N-acetylcysteine. Biochimica et Biophysica Acta (BBA) - General Subjects 1830(8):4117-4129. https://doi.org/10.1016/j.bbagen.2013.04.016 | Secondary/background source without original eligible NAC data | Review of NAC chemistry and biological activity; no original canine/feline dataset. |
| 28 | Sheffner AL (1963) The reduction in vitro in viscosity of mucoprotein solutions by a new mucolytic agent, N-acetyl-L-cysteine. Annals of the New York Academy of Sciences 106(2):298-310. https://doi.org/10.1111/j.1749-6632.1963.tb16647.x | No eligible canine/feline model | In vitro mucoprotein-viscosity experiment without canine/feline biological material. |
| 29 | Sies H (2015) Oxidative stress: a concept in redox biology and medicine. Redox biology, 4, 180–183. https://doi.org/10.1016/j.redox.2015.01.002 | Secondary/background source without original eligible NAC data | Conceptual review of oxidative stress; no original NAC intervention data. |
| 30 | Solis WA, Dalton TP, Dieter MZ, Freshwater S, Harrer JM, He L, Shertzer HG, Nebert DW (2002) Glutamate-cysteine ligase modifier subunit: mouse Gclm gene structure and regulation by agents that cause oxidative stress. Biochemical Pharmacology 63(9):1739-1754. https://doi.org/10.1016/s0006-2952(02)00897-3 | No eligible canine/feline model | Mouse glutamate-cysteine ligase study; no eligible canine/feline model. |
| 31 | Stopyra A, Sobiech P, Pomianowski A, Zarczynska K, Kaczorek E (2019) Nebulization as complementary therapy for dogs with respiratory tract infections. Insights in Veterinary Science 3(1):030-035. https://doi.org/10.29328/journal.ivs.1001017 | No direct NAC exposure | Canine nebulization study used saline-based supportive therapy; NAC was not administered. |
| 32 | Szakmany T, Hauser B, Radermacher P (2012) N-acetylcysteine for sepsis and systemic inflammatory response in adults. Cochrane Database of Systematic Reviews 2012(9):CD006616. https://doi.org/10.1002/14651858.CD006616.pub2 | Secondary/background source without original eligible NAC data | Human sepsis systematic review; no original canine/feline NAC dataset. |
| 33 | Tenório MCS, Graciliano NG, Moura FA, Oliveira ACM, Goulart MOF (2021) N-Acetylcysteine (NAC): impacts on human health. Antioxidants 10(6):967. https://doi.org/10.3390/antiox10060967 | Secondary/background source without original eligible NAC data | General human-health review of NAC; no original eligible veterinary data. |
| 34 | Tieu S, Charchoglyan A, Paulsen L, Wagter-Lesperance LC, Shandilya UK, Bridle BW, Mallard BA, Karrow NA (2023) N-Acetylcysteine and its immunomodulatory properties in humans and domesticated animals. Antioxidants 12(10):1867. https://doi.org/10.3390/antiox12101867 | Secondary/background source without original eligible NAC data | Review of NAC immunomodulation across species; no new original dataset. |
| 35 | Trzil JE (2019) Feline Asthma: Diagnostic and Treatment Update. Veterinary Clinics of North America: Small Animal Practice 50(2):375-391. https://doi.org/10.1016/j.cvsm.2019.10.002 | Secondary/background source without original eligible NAC data | Clinical review of feline asthma; no original NAC intervention dataset. |
| 36 | Üstün Alkan F, Bakirel T, Üstüner O, Yardibi H (2014) In vitro effects of doxorubicin and deracoxib on oxidative-stress-related parameters in canine mammary carcinoma cells. Acta Veterinaria Hungarica 62(3):372-385. https://doi.org/10.1556/AVet.2014.012 | No direct NAC exposure | Canine mammary-cell study of doxorubicin and deracoxib; NAC was not tested. |
| 37 | Uttamsingh V, Keller DA, Anders MW (1998) Acylase I-catalyzed deacetylation of N-acetyl-L-cysteine and S-alkyl-N-acetyl-L-cysteines. Chemical Research in Toxicology 11(7):800-809. https://doi.org/10.1021/tx980018b | No eligible canine/feline model | Biochemical acylase-deacetylation experiment without an eligible canine/feline model. |
| 38 | Vajdovich P (2008) Free radicals and antioxidants in inflammatory processes and ischemia-reperfusion injury. Veterinary Clinics of North America: Small Animal Practice 38(1):31-123. https://doi.org/10.1016/j.cvsm.2007.11.008 | Secondary/background source without original eligible NAC data | Veterinary review of free radicals and antioxidants; no original NAC dataset. |
| 39 | Vajdovich P, Kriska T, Mézes M, Szabó PR, Balogh N, Bánfi A, Arany-Tóth A, Gaál T, Jakus J (2005) Redox status of dogs with non-Hodgkin lymphomas: an ESR study. Cancer Letters 224(2):339-346. https://doi.org/10.1016/j.canlet.2004.11.037 | No direct NAC exposure | Measured redox status in canine lymphoma without direct NAC exposure. |
| 40 | Villar D, Buck WB, Gonzalez JM (1998) Ibuprofen, aspirin and acetaminophen toxicosis and treatment in dogs and cats. Veterinary and Human Toxicology 40(3):156-162. | Secondary/background source without original eligible NAC data | Toxicology review of analgesic toxicoses; no original NAC efficacy dataset. |
| 41 | Viviano KR, Lavergne SN, Goodman L, VanderWielen B, Grundahl L, Padilla M, Trepanier LA (2009) Glutathione, cysteine, and ascorbate concentrations in clinically ill dogs and cats. Journal of Veterinary Internal Medicine 23(2):250-257. https://doi.org/10.1111/j.1939-1676.2008.0238.x | No direct NAC exposure | Measured glutathione/cysteine/ascorbate in ill dogs and cats without NAC treatment. |
| 42 | Webb CB, Twedt DC, Fettman MJ, Mason G (2003) S-adenosylmethionine (SAMe) in a feline acetaminophen model of oxidative injury. Journal of Feline Medicine and Surgery 5(2):69-75. https://doi.org/10.1016/S1098-612X(02)00017-7 | No direct NAC exposure | Evaluated S-adenosylmethionine in feline acetaminophen injury; NAC was not tested. |
| 43 | Webster CRL, Cooper J (2009) Therapeutic use of cytoprotective agents in canine and feline hepatobiliary disease. Veterinary Clinics of North America: Small Animal Practice 39(3):631-652. https://doi.org/10.1016/j.cvsm.2009.02.002 | Secondary/background source without original eligible NAC data | Clinical hepatobiliary treatment review; no original eligible NAC dataset. |
| 44 | Wu G, Lupton JR, Turner ND, Fang YZ, Yang S (2004) Glutathione metabolism and its implications for health. The Journal of Nutrition 134(3):489-492. https://doi.org/10.1093/jn/134.3.489 | Secondary/background source without original eligible NAC data | Review of glutathione metabolism; no original canine/feline NAC dataset. |
| 45 | Zafarullah M, Li WQ, Sylvester J, Ahmad M (2003) Molecular mechanisms of N-acetylcysteine actions. Cellular and Molecular Life Sciences 60(1):6-20. https://doi.org/10.1007/s000180300001 | Secondary/background source without original eligible NAC data | Mechanistic review of NAC actions; no original eligible canine/feline dataset. |

*Abbreviations: NAC, N-acetylcysteine; CKD, chronic kidney disease.*
